# Context-dependent plant–bird interactions shape polychory across the antagonism–mutualism continuum

**DOI:** 10.1038/s42003-026-10142-x

**Published:** 2026-04-28

**Authors:** Dailos Hernández-Brito, Fernando Hiraldo, Jaume Izquierdo-Palma, José L. Tella, Martina Carrete

**Affiliations:** 1 Department of Conservation Biology and Global Change, Estación Biológica de Doñana (CSIC), Seville, Spain; 2 Department of Physical, Chemical and Natural Systems, Universidad Pablo de Olavide, Seville, Spain

**Keywords:** Evolutionary ecology, Plant ecology, Behavioural ecology, Ecosystem services

## Abstract

Animals feeding on plants may benefit them through seed dispersal or harm them through seed predation, causing net interaction outcomes along an antagonism–mutualism continuum that remains poorly understood. We systematically monitored 6,012 foraging visits of 25 bird species interacting with 40 plant species over a full annual cycle. Interaction outcomes (seed predation, fruit defleshing, and seed dispersal) and dispersal mechanisms (endozoochory, stomatochory, and epizoochory) were shaped by fruit traits, bird feeding strategies, and fruit-to-bird size ratios. However, nearly all bird species (and 10% of individual foraging visits) combined multiple interaction outcomes, seed predators often acting as effective non-endozoochorous dispersers. This flexibility generated polychory in 50% of plant species, while 47.5% were dispersed by birds solely through stomatochory or epizoochory, mechanisms overlooked by classical dispersal syndromes. Our findings portray birds as dynamic, context-dependent agents of seed fate, highlight the need to integrate trait-based and behavioural perspectives in seed dispersal ecology.

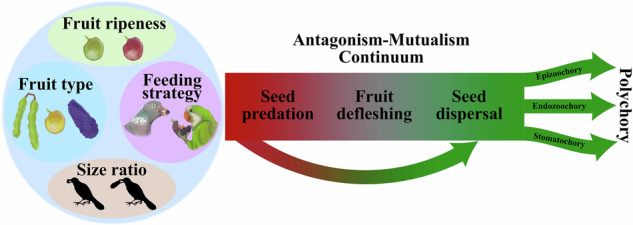

## Abstract (web summary)

Bird–plant foraging interactions show many birds act as both seed predators and dispersers, using multiple modes (polychory). Seed fate depends on traits, context, and flexible feeding behavior.

## Introduction

Plant–animal interactions encompass a broad range of antagonistic and mutualistic relationships that are fundamental to community assembly, ecosystem functioning and biodiversity preservation across all scales^1,2^. Among the most extensively studied of these interactions is seed dispersal, a critical mutualism in which plants benefit from enhanced gene flow and colonisation of new habitats^1–3^, while animals gain nutritional rewards from consuming and digesting the fruit pulp^4,5^. However, plants attract a functionally diverse array of consumers, some of which act as antagonists by destroying seeds^6^. To navigate this trade-off between embryo protection and effective seed dispersal, plants have evolved a range of defensive strategies, such as hard seed coats (physical barriers^7^) or chemical deterrents (chemical defences^8,9^). The level and characteristics of such defences often change with fruit ripeness, dynamically influencing the balance between antagonisms and mutualisms^7,10,11^.

Birds play diverse functional roles in ecosystems, acting as both plant antagonists (e.g. predators of flowers and seeds) and mutualists (e.g. seed dispersers and pollinators)^6,12^. Yet the factors governing the outcomes of plant–bird interactions remain under debate^13–16^. Traditional frameworks have emphasised bird feeding strategies, viewing as legitimate dispersers only species that swallow fruits whole (i.e. gulpers^17^), digest the pulp, and later excrete or regurgitate intact seeds (i.e. endozoochory^18^). Conversely, birds that grind, crush or peck seeds (i.e. grinders, crushers and peckers^17^) have typically been classified as antagonists^6^. This dichotomy is further reinforced by the long-held assumption that propagule (i.e. seed and/or fruit) morphology rigidly dictates interaction outcomes and dispersal modes^18^. As a result, research has disproportionately focused on endozoochorous dispersal by frugivorous birds swallowing fleshy fruits^12,16,19–21^, with particular emphasis on the fruit-bird size matching constraints^12–14,22^. However, mounting evidence reveals that this morphologically deterministic view oversimplifies the real scenario. Many birds previously classified as seed predators frequently disperse seeds through less-recognised mechanisms, such as stomatochory (active transport of viable propagules away from the mother plant^23–26^), synzoochory (similar to stomatochory but involving seed caching^27–29^) or epizoochory (transport of viable seeds attached to bird bodies^30,31^). Similarly, many endozoochorous species also engage in pulp feeding or seed predation^12^, further blurring the distinction between antagonists or mutualists and supporting the existence of a continuum of ecological interactions in which roles can shift or overlap depending on context. Moreover, this continuum may also favour the prevalence of polychory (i.e. the coexistence of multiple dispersal mechanisms^18^), which emerges from the interplay between trait-based potential^18^, and the behavioural flexibility of consumers during foraging^32^, all shaped by contextual variability^33,34^.

Despite its ecological significance, the antagonism–mutualism continuum in seed predation and dispersal remains understudied in birds. Existing research is taxonomically biased, with a disproportionate focus on a few avian groups, such as corvids^27,28^ and other passerines^30,35^, parrots^10,11,23–25,31,36^, and pigeons^37,38^. This narrow taxonomic scope, coupled with the logistical challenges of collecting detailed observational data in natural systems, has led to an oversimplified understanding of plant–bird interactions^14,15,39–43^ that overlook intraspecific variation, an important source of ecological complexity. Recently, individual-based approaches have begun to reveal the ecological and evolutionary relevance of both intra- and inter-individual variability^32,44^, although our ability to scale up these dynamics to community and ecosystem levels remains limited^45–47^.

**Fig. 1:**
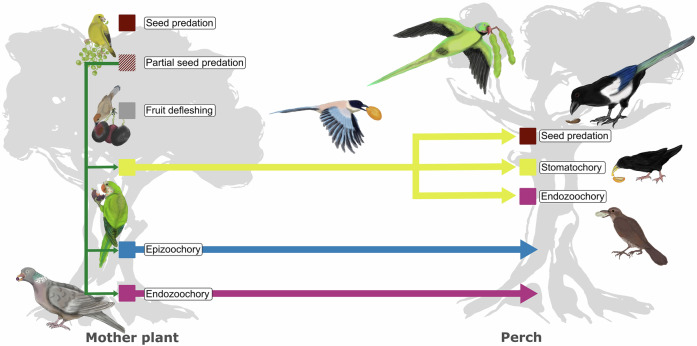
Birds interact with plants along a dynamic antagonism–mutualism continuum. At the mother plant, some birds may consume fruits without damaging seeds by removing only the pulp (fruit defleshing, grey square) or by swallowing seeds intact (endozoochory, purple symbols). Handling viscous pulp may also cause seeds to adhere to plumage (epizoochory, blue symbols). Other birds act as seed predators (dark red square), destroying embryos by crushing seeds with strong beaks, digesting them with powerful gizzards, or pecking them before protective coats harden. In multi-seeded fruits, seed predators may leave some seeds intact (partial seed predation, barred dark red square) and transport them away through different zoochorous mechanisms (green arrows). At distant perches, birds may disperse seeds passively by endozoochory (purple symbols) or epizoochory (blue symbols), actively by stomatochory (yellow symbols), or destroy them, thereby nullifying dispersal (dark red square). Illustrations by Dailos Hernández-Brito.

Here, we examine avian foraging activity across a diverse assemblage of fruiting plants to better understand the ecological and evolutionary factors shaping plant–bird interactions—specifically seed predation, fruit defleshing and seed dispersal—and their associated dispersal mechanisms (i.e. endozoochory, epizoochory and stomatochory) (Fig. 1). We anticipate that interaction outcomes and dispersal pathways will be primarily influenced by species-level traits, in particular bird foraging strategies and fruit types, and size compatibility and constraints between partners^13,14,22,25,29,48,49^, all shaped by shared evolutionary histories^15,25^. For instance, we expect dry fruits to experience higher seed predation^48^, whereas fleshy fruits will be more likely swallowed whole and dispersed via endozoochory by gulpers and grinders. However, we also predict that some dry-fruited seeds, especially when handled by smashers or peckers, as well as large fleshy fruits that exceed a bird’s swallowing capacity, may still be successfully dispersed through stomatochory or epizoochory^26,31,36^. Importantly, we further propose that these deviations from typical dispersal patterns may increase in frequency under specific ecological conditions. For example, changes in fruit ripeness may modify sensory and nutritional cues, thereby altering bird preferences and handling behaviours, which ultimately influence their ecological roles. These apparent deviations from the traditionally expected patterns may become more prevalent under certain ecological contexts. Thus, we predict that unripe fruits will experience higher rates of seed predation and lower rates of dispersal via endozoochory compared to ripe fruits. However, unripe fruits may still be dispersed through alternative mechanisms, such as stomatochory and epizoochory, as these pathways are often linked to predation processes carried out by species with strong beaks and/or feet, or involve fruits with viscous pulp or resins. Additionally, we predict that species do not occupy fixed positions along the antagonism–mutualism continuum but instead exhibit dynamic, context-dependent roles^32,47^. A key question arising from this intraspecific variability is whether it results from behavioural plasticity—where each individual is capable of shifting flexibly among roles such as seed predator, fruit deflesher, or seed disperser using different dispersal mechanisms—or from the coexistence of individuals with different specialisations, where each bird individual systematically operates the same role and dispersal mechanism. Disentangling the influence of fixed species-level traits from individual-level variation will be essential to deepen our understanding of the complexity and dynamism inherent in plant–bird interactions, as well as their broader ecological and evolutionary implications.

## Results

We accumulated 402 h of field observation and recorded 6012 foraging bird visits (i.e. each visit by a bird, regardless of whether it was the same individual, as birds were not individually marked, see 'Methods') from 25 species (body mass 11.2–490 g; Table S1) interacting with 21,906 fruits from 578 individual plants of 40 species (16 dry-fruited, 14 drupaceous and 10 berry-like species; range of fruit sizes: 4.2–300 mm; Tables S2 and S3). The median visit duration was 83 s (range: 2– 300 s, see 'Methods') and feeding intensity varied substantially across individual plants (range: 1–84 visiting birds per plant; median = 7.5) and individual bird visits (1–124 fruits consumed per bird visit; median = 2). Approximately 67% of the consumed fruits were ripe.

### Drivers of plant–bird interactions and dispersal mechanisms

**Fig. 2:**
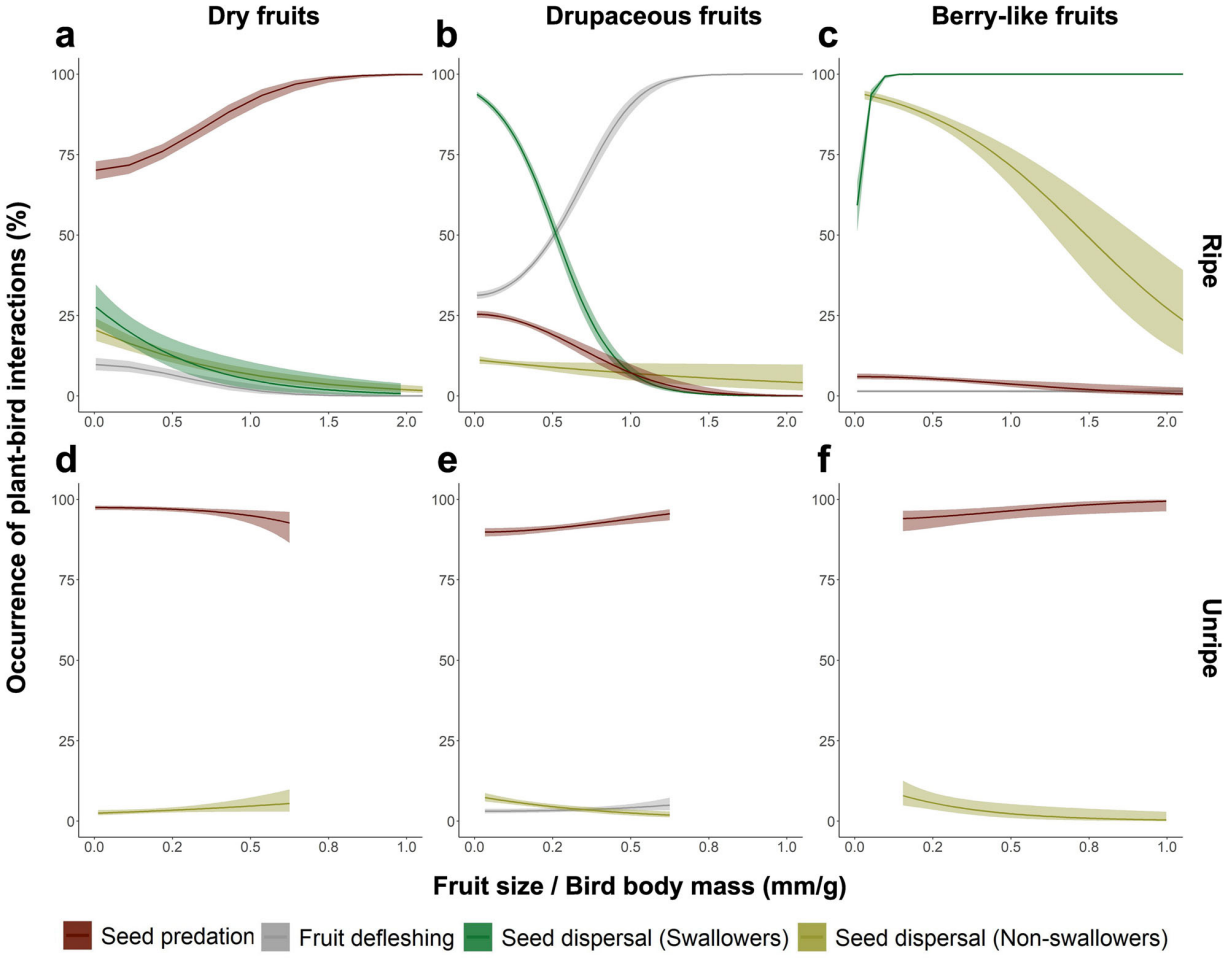
Probabilities of seed predation, fruit defleshing and seed dispersal under different factors. For ripe (**a**–**c**) and unripe (**d**–**f**) fruits, across different fruit types: dry (**a**, **d**), drupaceous (**b**, **e**) and berry-like (**c**, **f**). Probabilities are modelled as functions of fruit size relative to the body mass of interacting birds. Bird feeding strategy (i.e. swallower and non-swallower) was only shown for seed dispersal probabilities. Probabilities are derived from significant fixed effects in the best-fitting models (Table S4). Lines represent predicted values, with shaded areas indicating 95% confidence intervals.

Seed predation was the most frequent plant–bird interaction (48.2% of fruits), followed by seed dispersal (35.6%) and fruit defleshing (16.1%) (Data S1). Phylogenetically controlled models revealed that fruit fate (i.e. whether a fruit was defleshed or its seeds were predated or dispersed) was shaped by fruit type, ripeness, bird feeding strategy and the fruit-to-bird size ratio (Figs. 2 and S1, Table S4). Unripe fruits were more prone to seed predation, while ripe fruits showed patterns that varied with fruit type and size relative to bird body mass: large dry fruits were especially vulnerable, as were smaller drupaceous and berry-like fruits. In contrast, fruits that were large relative to bird size, ripe and drupaceous were more commonly defleshed (Fig. 2a–c). Bird feeding strategy had a consistent effect, with swallower birds being less likely to predate seeds or deflesh fruits (Table S5). Seed dispersal was more likely for fruits that were small relative to bird size and either dry or drupaceous fruits, regardless of bird feeding strategy (Fig. 2a, b), as well as for berry-like fruits that were large relative to bird size when consumed by swallowers (Fig. 2c). However, seed dispersal probability dropped when non-swallowers that were small relative to fruit size fed on unripe fleshy fruits (Fig. 2d–f).

**Fig. 3:**
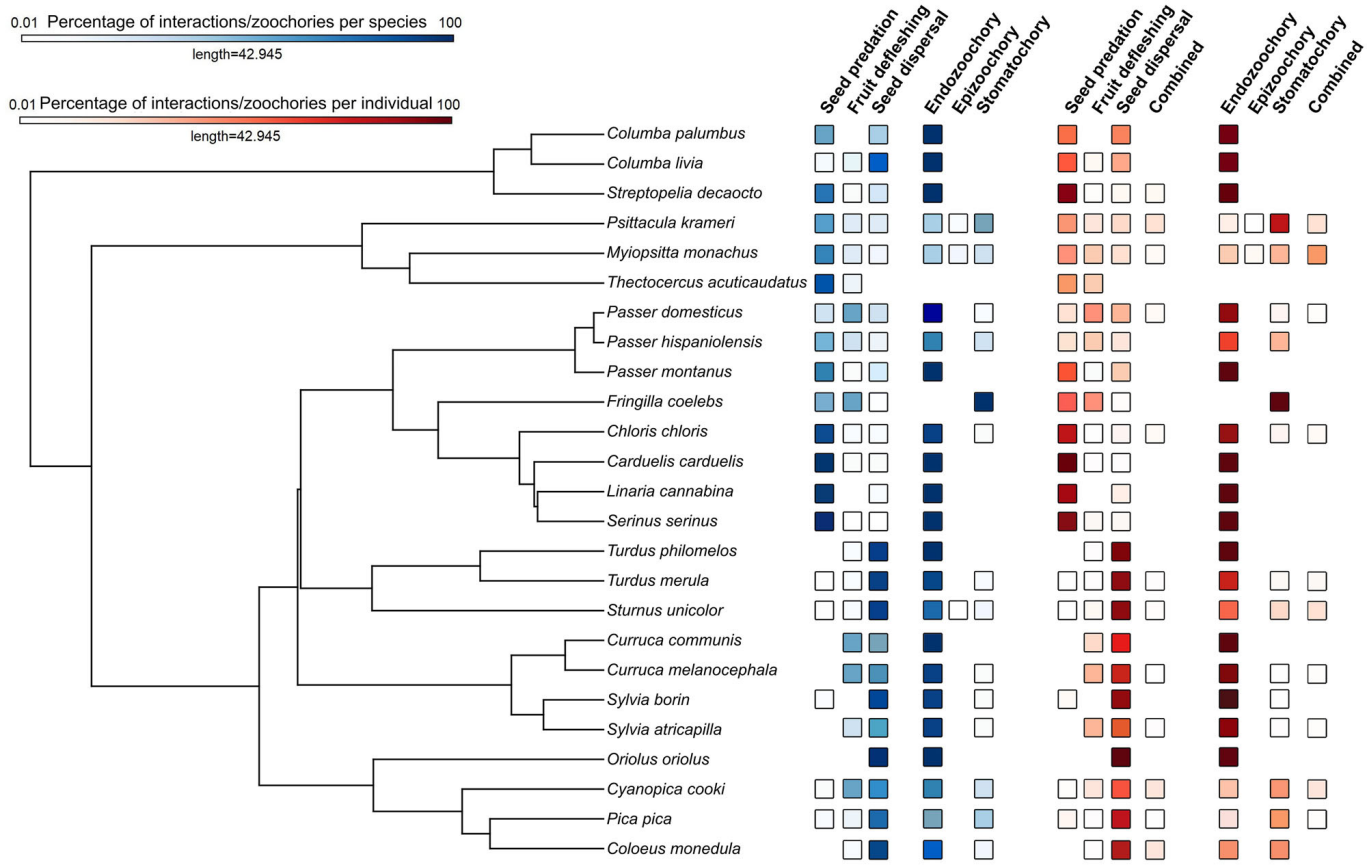
Percentages of recorded plant–bird interactions (seed predation, fruit defleshing and seed dispersal) and seed dispersal mechanisms (endozoochory, epizoochory and stomatochory) across bird species (blue squares) and individuals (red squares). The “combined” category denotes the percentage of individuals performing multiple interaction types or dispersal mechanisms concurrently. Lighter-coloured squares indicate values approaching, but not reaching, zero.

**Fig. 4:**
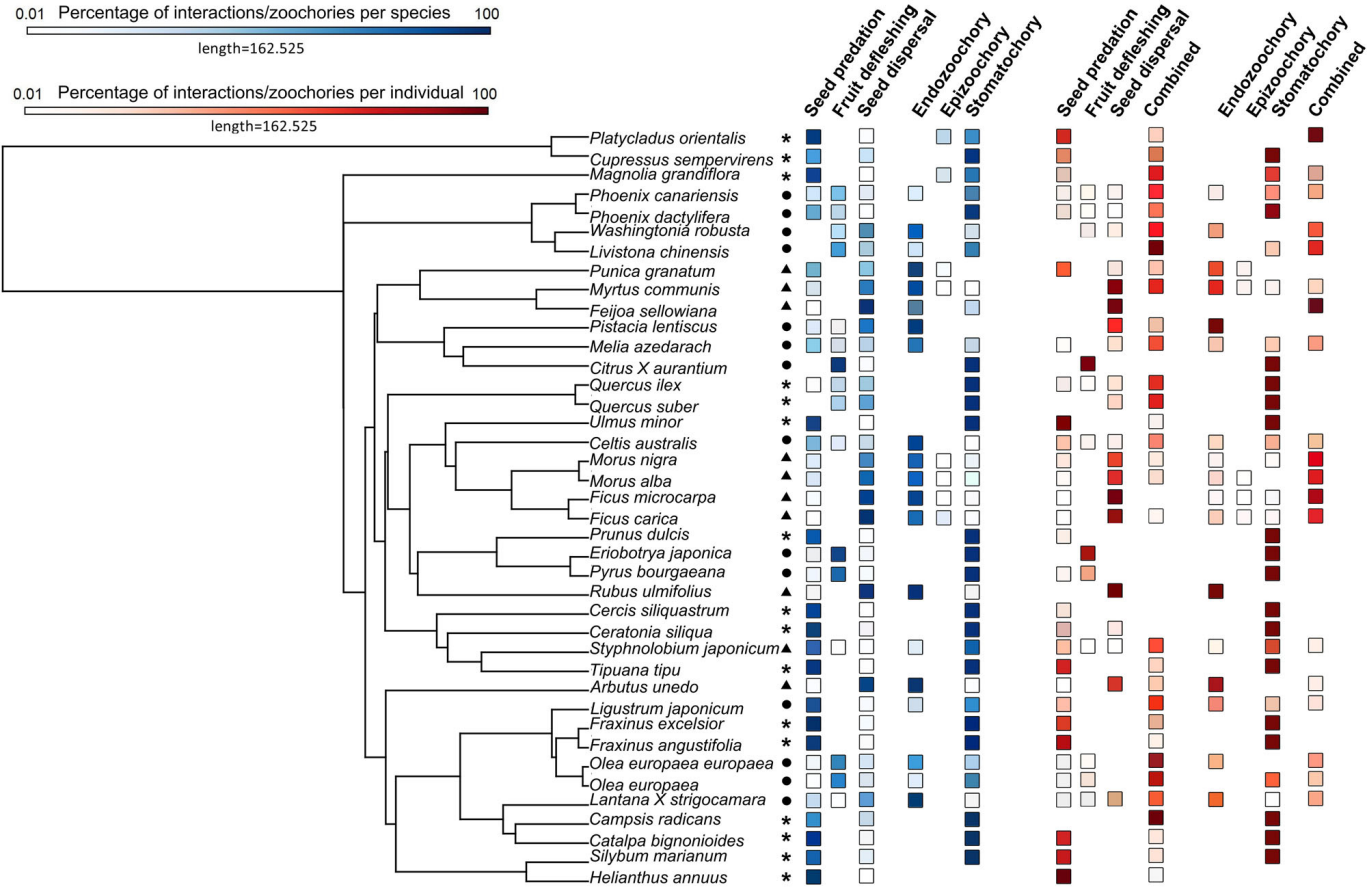
Percentages of recorded plant–bird interactions (seed predation, fruit defleshing and seed dispersal) and seed dispersal mechanisms (endozoochory, epizoochory and stomatochory) across plant species (blue squares) and individuals (red squares). The “combined” category denotes the percentage of individuals exhibiting multiple interaction types or dispersal mechanism concurrently. Lighter-coloured squares indicate percentages values approaching, but not reaching, zero. Symbols denote fruit types: dry (asterisk), drupaceous (circle) and berry-like (triangle).

Plant–bird interactions showed weak phylogenetic signals in both birds (range *λ*_birds_: <0.01–0.04) and plants (range *λ*_plants_: 0.01–0.15), with small variance explained by species identity and phylogeny, except in the case of seed predation (Table S4). Functionally, most bird species (96%) performed multiple roles, acting as seed predators and/or dispersers and/or fruit defleshers (Fig. 3 and Data S1). Some individuals even switched roles within a single foraging bout, predating some seeds while dispersing others via endozoochory or stomatochory (Table S5). Similarly, only 30% of plant species were predominantly (i.e. > 90% of observed fruits) either predated or dispersed (Fig. 4 and Data S1).

**Fig. 5:**
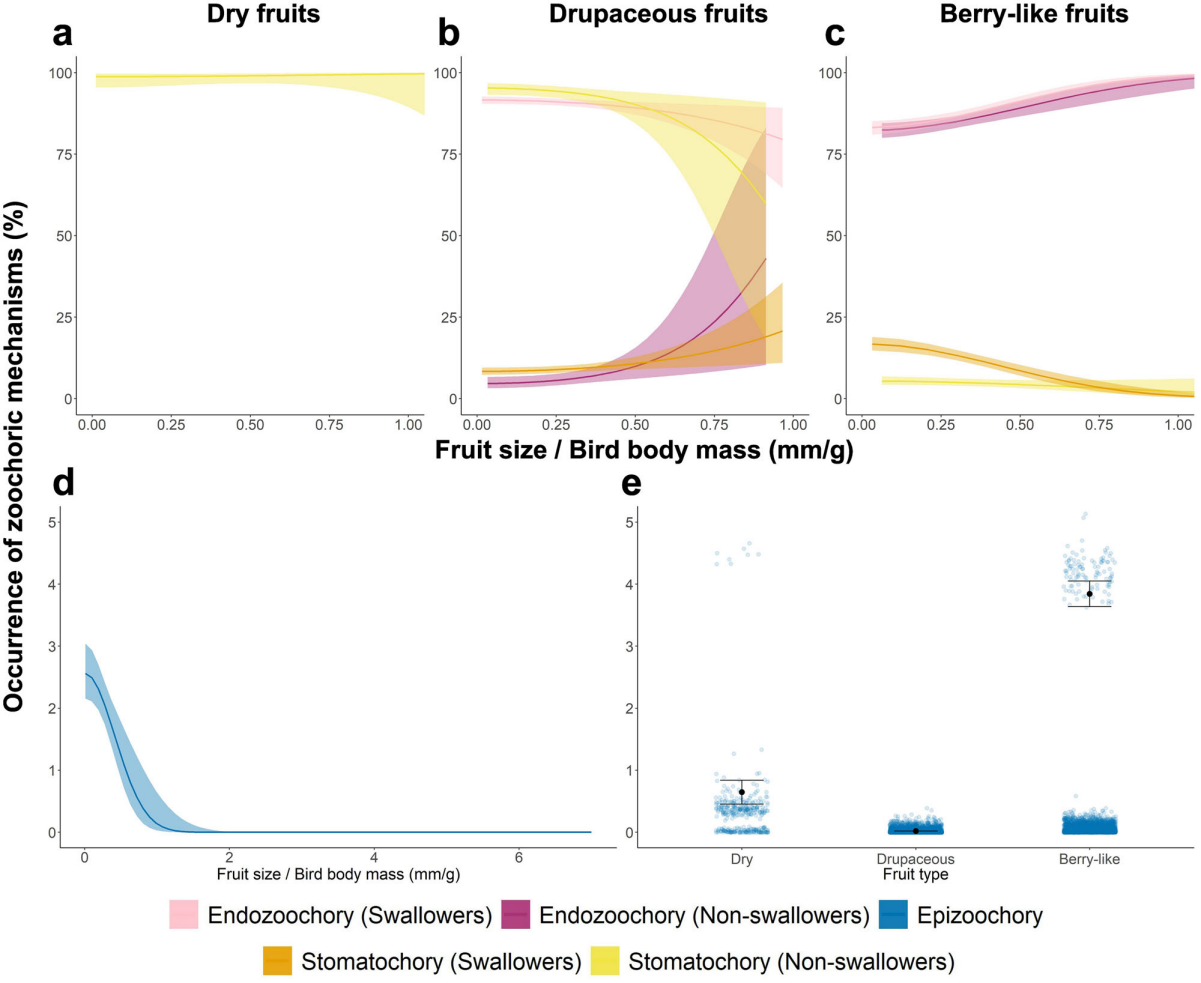
Probabilities of seed dispersal by different mechanisms under key factors. Via endozoochory and stomatochory by swallowing and non-swallowing bird species for dry (**a**), drupaceous (**b**) and berry-like (**c**) fruits, shown as a function fruit size relative to the body mass of interacting birds. Seed dispersal via epizoochory was influenced independently by the fruit-to-bird size ratio (**d**) and fruit type (**e**), with no interaction between these variables. Predictions are derived from significant fixed effects in the best-fitting models (Table S6). Lines and dots represent predicted values, with shaded areas (**a**–**d**) and whiskers on dots (**e**) indicating 95% confidence intervals.

Among dispersal mechanisms, endozoochory was dominant (77.9% of dispersed fruits), followed by stomatochory (20.1%) and epizoochory (2.0%) (Data S1). The relative use of each mechanism depended strongly on fruit type, bird feeding strategy and fruit-to-bird size ratio (Figs. 5 and S2, Table S6). Endozoochory was more frequent for small drupaceous fruits consumed by swallowers of relatively small size, whereas non-swallowers showed higher endozoochory rate when handling fruits that were large relative to their body mass (Fig. 5b). For berry-like fruits, endozoochory increased consistently with fruit-to-body-mass ratio across both feeding strategies (Fig. 5c). Thus, for drupaceous fruits, swallowers show higher endozoochory probabilities when they handled small fruits relative to their body mass, whereas non-swallowers show higher probabilities when fruits were large relative to body mass. For berry-like fruits, higher fruit-to-body-mass ratios consistently favour endozoochory across both feeding strategies. In contrast, stomatochory occurred more frequently when dry and drupaceous fruits that were large relative to bird size were handled by non-swallowers (Fig. 5a, b) as well as when berry-like fruits were small relative to bird body mass (Fig. 5c). Notably, over 35% of stomatochory events involved the transport of multiple fruits (mean = 6.69, range: 2–45) from single or multiple infructescences (Table S5). Epizoochory, although rare, typically involved small berry-like fruits consumed by relatively large birds (Fig. 5d, e). Overall, these patterns indicate that size constrains between fruits and birds plays an important role in shaping dispersal mechanisms, although bird feeding strategies, fruit ripeness and fruit type also contribute to this process.

Dispersal mechanisms, like interaction outcomes, displayed weak phylogenetic signals in both plants (range *λ*_plants_: 0.03– 0.04) and birds (range *λ*_birds_: 0.01–0.06). Yet, species identity explained slightly more variance than ecological predictors alone in models of endozoochory and epizoochory (Table S6). However, both bird and plant species frequently engaged in multiple dispersal strategies. Thirteen out of 24 bird species combined endozoochory with stomatochory (Fig. 3 and Data S1), while 20 out of 40 plant species relied on more than one dispersal mechanisms (Fig. 4 and Data S1). Compared to traditional assumptions (Tables S2 and S3), polychory was strikingly more common in our study. Of the 28 plant species formerly considered endozoochorous, 89.3% were also dispersed by stomatochory and 25.0% via epizoochory. Interestingly, eight of these species showed no evidence of endozoochory during our observations. Additionally, all eight anemochorous species, the single myrmecochorous species and all three synzoochorous species in our sample were also dispersed through stomatochory. Polychory was slightly more frequent in non-native species, occurring in 66.7% of birds and 100% of plants, compared to 45.5% of birds and 87.5% of native plants.

### Variability in plant–bird interactions and dispersal mechanisms

Despite clear associations with bird feeding strategies, fruit traits and size compatibility, plant–bird interactions were highly variable and often shifted along the antagonism–mutualism continuum (Fig. 1). Among the 3861 multi-seeded fruits consumed on the mother plant (1655 dry, 1154 drupaceous and 1052 berry-like), 89% had all seeds destroyed. In the remaining 11%, variable numbers of seeds were successfully dispersed by stomatochory (49.3%), endozoochory (49.04%) or, less frequently, epizoochory (1.68%) (Table S5). Additionally, another 1571 non-multi-seeded fruits were plucked and transported away from the mother plant, with seeds subsequently either discarded intact (stomatochory, 62.3%), predated (19.9%) or swallowed (endozoochory, 17.8%) at distant perches (Fig. 1).

**Fig. 6:**
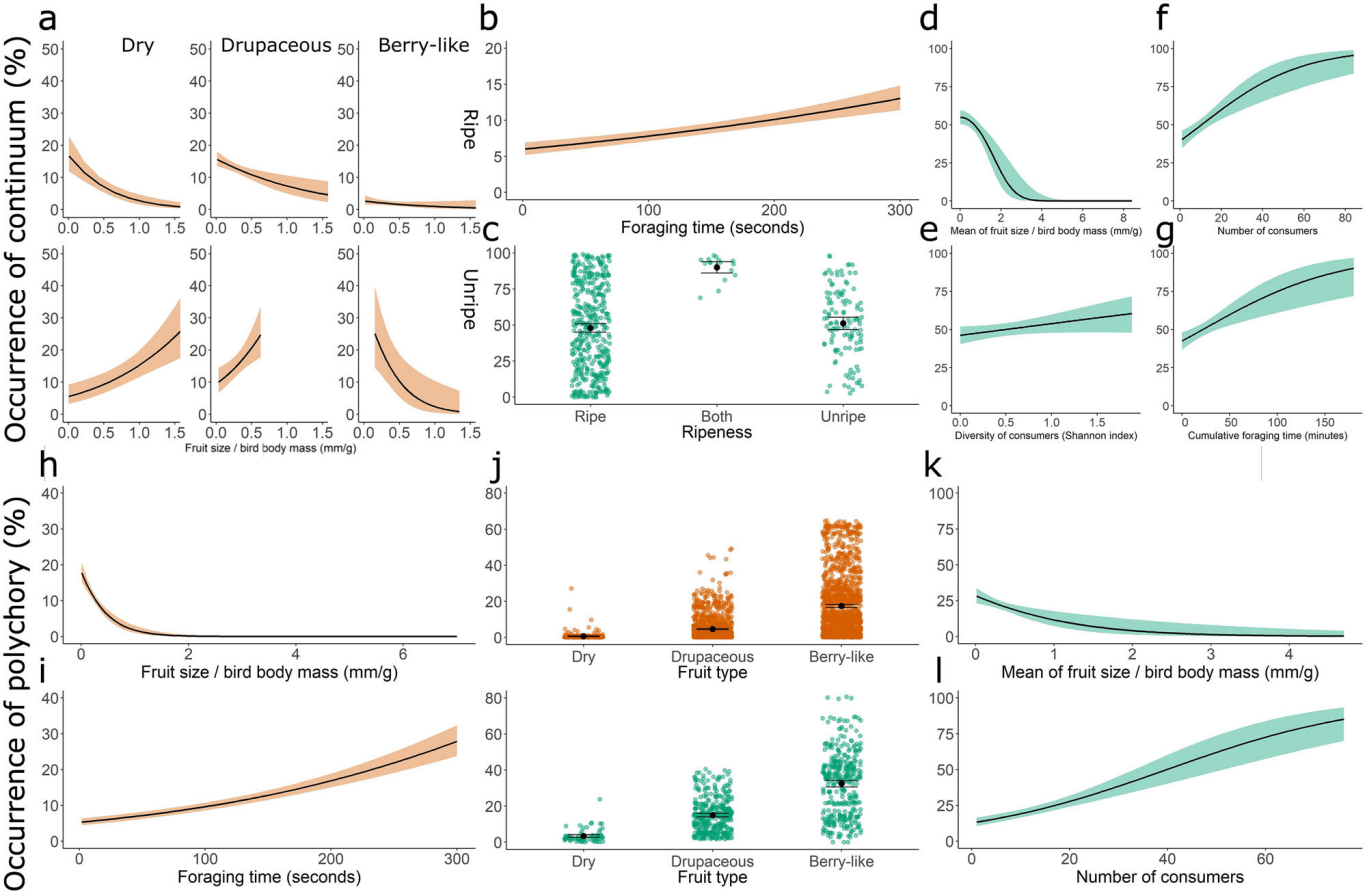
Factors influencing the probability that individuals display dynamic processes. Individual plants (green) or individual visits of birds (orange) engage in (**a**–**g**) interactions along the antagonism–mutualism continuum (seed predation, fruit defleshing and/or seed dispersal) and **h**–**l** polychory (endozoochory, epizoochory and/or stomatochory) during observation periods (30 min for plants, up to 5 min for birds). Predictions are derived from significant fixed effects in the best-fitting models (Tables S8 and S10). Lines and dots represent predicted values, with shaded areas and whiskers on dots indicating 95% confidence intervals.

During the observation period (up to 5 min), most birds primarily acted as predators, defleshers or dispersers. However, nearly 10% of visits involved multiple roles, combining seed predation and dispersal (3.1%), fruit defleshing and seed predation (1.0%) or seed predation and dispersal (4.9%). A small fraction (0.1%) encompassed all three interactions within a single foraging bout (Fig. 3 and Table S7). These mixed behaviours along the antagonism–mutualism continuum were more frequently observed during prolonged visits on plants bearing small drupaceous and berry-like unripe fruits that were small relative to bird size (Fig. 6a, b and Table S8). Individual plants showed greater variation: while half experienced only one interaction type, the remaining received mixed interactions (Fig. 4 and Table S9). Individual plants receiving combinations of interactions were those bearing a mixture of unripe and ripe fruits that were small relative to the bird’s size and attracted numerous and taxonomically diverse avian consumers engaging in extended foraging bouts (Fig. 6c–g and Table S10).

Patterns of dispersal mirrored this complexity. Although 90% of individual bird visits involved a single strategy, approximately 10% included multiple mechanisms, most commonly combinations of endozoochory and stomatochory (6.0%), endozoochory and epizoochory (4%) and, rarely, all three (0.2%) (Fig. 3 and Table S7). The likelihood of employing multiple dispersal mechanisms increased during longer individuals foraging bouts and when birds consumed berry-like fruits that were small relative to their body mass (Fig. 6h–j and Table S8). This variability was more pronounced among individual plants. While 62% of individuals were dispersed via a single mechanism, 38% experienced polychory. Among these, 27.8% were dispersed by both endozoochory and stomatochory, and 5.5% by all three mechanisms (Fig. 4 and Table S9). Polychory was common in plants bearing berry-like fruits, small fruits relative to bird size, and receiving large numbers of foraging birds (Fig. 6j–l and Table S10).

Phylogenetic effects were weak (range *λ*_birds_: <0.01–0.06, range *λ*_plants_: <0.01–0.01), and species identity did not explain variation in the likelihood that individuals bird visits combined multiple interaction outcomes or dispersal mechanisms. However, the identity of the plant species on which birds foraged accounted for a substantial proportion of the observed variance (Tables S8 and S10).

## Discussion

Our comprehensive, year-round observations of diverse bird species foraging on fruits of varying types, sizes and ripeness stages reveal that plant–bird interactions are far more variable and context-dependent than traditionally assumed. Rather than fitting neatly into static categories, these interactions exist along a dynamic continuum characterised by frequent shifts in interaction outcomes and species’ ecological roles. Consistent with findings from other ecological systems, biotic interactions are often conditional and can change over time, with species oscillating between roles traditionally labelled as mutualistic, antagonistic, or neutral depending on ecological context and scale^50–52^. Accumulating evidence of this complexity challenges paradigms that categorise species strictly as antagonists or mutualists, exposing critical limitations in current ecological frameworks. A key implication of our results is that species’ ecological roles cannot be reliably predicted solely based on evolutionary relatedness or species identity. Instead, multiple interaction types and dispersal mechanisms often occur simultaneously, sometimes even within individual plants and during short feeding visits by birds. For instance, previous analyses of flower-visitor assemblages have shown that the same bird or insect species can function as legitimate pollinators under certain conditions but act as antagonists (e.g. nectar robbers or floral predators) under others^51^. Such behavioural plasticity reflects shifting cost-benefit balances and context-dependent fitness effects documented across taxa and systems^53^. Together, these findings call into question the validity of binary classification systems—such as antagonist vs. mutualist^14,19,39,40^—and trait-based syndromes^18,42^ that have facilitated standardised global data collection (see for reviews^54,55^), suggesting that these simplifications obscure the true functional diversity of species and interactions and may conceal critical aspects of ecosystem dynamics.

### Drivers of plant–bird interactions and seed dispersal mechanisms

Understanding the outcomes of plant–bird interactions—and their consequences for seed dispersal—requires more than evaluating species traits in isolation. Instead, it demands attention on the interplay between morphological, behavioural and ecological factors, a dimension that remains rarely explored due to its inherent complexity.

Fruit-bird size matching has long been considered a fundamental determinant of plant–bird interactions, as it constraints whether a bird can physically handle, ingest or transport a fruit, ultimately shaping the type and effectiveness of seed dispersal^13,14,22,25,29,49,56,57^. Although recent studies have challenged the rigidity of this relationship by showing that some birds can consume fruits larger than their gape^49^, our findings largely support the ecological importance of size compatibility. Importantly, our size-ratio approach differs from the trait-matching framework commonly used in the literature, which typically quantifies similarity between fruit and bird sizes under gape size constraints^56,57^. Instead, our metric captures the tendency for birds to consume fruits that are small relative to their body size. In this context, size ratios support that fruits that are small relative to bird size are typically swallowed whole and dispersed endozoochorously, while seeds of larger fruits are more often predated or handled, facilitating defleshing and the emergence of alternative seed dispersal modes, such as stomatochory. Notably, size-compatibility is not a strict threshold: interactions near the trait-matching limit frequently yield mixed outcomes, even within the same individual plant, particularly when fruits occur at different ripeness stages. These size-mediated interactions have important consequences for plant eco-evolutionary strategies and subsequent seedling recruitment. Plants producing larger seeds generally experience higher rates of seed predation than those with smaller seeds, which may favour strategies aimed at attracting animals capable of handling and dispersing seeds through pathways other to endozoochory. By promoting seed movement away from the mother plant rather than reliance on dormancy or local persistence, such strategies can reduce density-dependent post-dispersal seed predation^58^. However, size matching alone does not fully determine interaction outcomes. Consumer fruit selection is also shaped by trade-offs between nutritional rewards and energetic costs^34^. While larger fruits often provide greater energetic returns, they also require increased manipulation effort, whereas smaller fruits, though less profitable individually, tend to be more abundant and easier to ingest^34^. Together, consumer morphological constraints, handling capacities and plant investment strategies interact to mediate these trade-offs, such that plant–animal interactions emerge from a dynamic balance between mechanical feasibility and energetic cost-benefit considerations, rather than from size matching alone.

Fruit type has traditionally been used to infer ecological roles and define dispersal syndromes, often constraining research opportunities to assumed mutualistic or antagonistic interactions. However, our data and emerging studies^49,54,55^ reveal a broader spectrum of viable interactions and dispersal pathways than these classical models predict. Dry fruits, where nutritional value is concentrated in the seeds, have been largely associated with seed predation by birds^7^, with dispersal opportunities attributed mostly to mammals and corvids^6,27,59^. Our results partly support this view but demonstrate that the reality is more nuanced. Unripe dry fruits were more frequently predated, particularly by non-swallowers, many of which acted as dual agents^49^, damaging some seeds but dispersing others, often when ripe, through stomatochory or epizoochory^26,31,54,55^. This dual role is particularly important in multi-seeded fruits, where dispersal of some seeds can still enhance overall reproductive success (Fig. 1). These patterns are consistent with observations in primates^60^ and align with extensive evidence from parrots^10,11,23–25,31^, waterbirds^54^ and corvids^26–28^, reinforcing the broader role of birds as legitimate dispersers of dry-fruited plants.

Fruit ripeness further shapes interaction outcomes by altering both the physical defences and chemical composition of fruits and seeds. Our hypothesis that unripe fruits suffer greater seed predation and lower endozoochorous dispersal was supported by observations showing swallowing and non-swallowing birds frequently damaging unripe seeds^10,60^. In addition, early removal of unripe fruits is often ineffective for dispersal, as they may not complete development once detached from the mother plant. Many plants mitigate this risk through chemical deterrents^9^, such as phenolic compounds in *Olea spp*^61^ and *Livistona chinensis*^62^. Still, some birds can overcome these defences through detoxification mechanisms^8,9,63^ or behavioural adaptations like geophagy or regulated ingestion^63^, even dispersing unripe fruits via stomatochory and epizoochory. As fruits ripen, seeds acquire more robust coats and the pulp becomes more palatable, increasing the probability of effective dispersal. Small ripe fleshy fruits were generally swallowed whole, while larger berry-like fruits were consumed in pieces, facilitating endozoochory^12,13,49^. In contrast, large ripe drupaceous fruits that exceeded avian gape size were typically defleshed, especially by non-swallowers^49^. While defleshing has often been seen as functionally neutral or unknown for plants^4,14,15,41^, mounting evidence points to its complex fitness consequences^8,64,65^. Our data show that defleshing frequently co-occurs with other interactions, mainly in plants bearing ripe fruits. Birds that carry fruits by stomatochory to deflesh them at distant perches can leave behind intact, pulp-free seeds (Fig. 1), potentially enhancing germination success by reducing exposure to pathogens and predators^66^.

We found compelling evidence that bird species do not occupy fixed ecological roles but exhibit substantial behavioural plasticity, with individuals shifting flexibly between seed predation, fruit defleshing and seed dispersal within a single foraging bout. This dynamic role switching extends to the use of multiple dispersal mechanisms, illustrating that intraspecific variation is critical for understanding interaction outcomes. In line with these results, our analyses revealed a weak phylogenetic signal in both interaction outcomes and dispersal mechanisms. While this should be interpreted cautiously—estimates are highly sensitive to phylogenetic tree topology (e.g. branch lengths and species representation) and may be unreliable when derived from relatively small species sets^67^—it may reflect the key insight that plant–bird interactions are shaped not only by the evolutionary histories of the species involved but also by the ecological contexts in which they occur. Many of the traits we examined or interpreted—fruit type and secondary metabolite clades in plants^68,69^ and feeding strategies and digestive adaptations in birds^70^—are evolutionarily conserved. Yet, the outcomes of interactions (i.e. whether a seed is destroyed or dispersed and how) often diverge from what phylogenetic expectations would suggest. This mismatch underscores that is not traits per se but their functional interaction, modulated by other, less-studied contextual variables like fruit ripeness at the moment of consumption, availability of alternative resources, intra- and inter-specific competition, predation risk and environmental conditions, that can further alter foraging behaviour^33,34^ and determine the direction of interactions and the dispersal pathways. Thus, the weak phylogenetic signal we observed is not inconsistent with trait conservatism, but rather reflects the inherently dynamic and contingent nature of mutualisms and antagonisms.

### Seed dispersal: moving beyond classical dispersal syndromes

Our findings emphasise the widespread and understudied role of non-endozoochorous dispersal mechanisms, particularly stomatochory and epizoochory, which were prominent in many plant and bird species and frequently co-occurred with endozoochory. While endozoochory dominated in small drupaceous fruits (swallow whole) and large berry-like fruits (pecked apart) consumed by swallowers, many plant species typically assumed to be endozoochorous were also dispersed through alternative modes. Specifically, seeds of large drupaceous and dry fruits were primarily dispersed via stomatochory by non-swallower birds, while seeds of small berry-like fruits were commonly dispersed via epizoochory. Remarkably, stomatochory was the sole dispersal mechanism for 47.5% of plant species in our study, yet it remains understudied^26^, likely due to its association with seed predation rather than classical dispersal syndromes. Traditional frameworks for seed dispersal have largely relied on propagule morphology to infer dispersal mechanisms^18^. Within this paradigm, fleshy-fruited seeds were typically associated with endozoochory^12,13,19–21,39^, while dry-fruited seeds were thought to depend on abiotic vectors like wind (anemochory) or biotic agents as ants (myrmecochory) or seed-catching animals as rodents or corvids (synzoochory^18,27,28^). This model, however, oversimplifies the complex and diverse interactions between plants and animals. Importantly, this neglect may arise from fundamental differences in how stomatochory operates. Unlike endozoochory, which involves ingestion of nutritious pulp and indigestible parts^71^, stomatochory entails active fruit handle, often away from the mother plant, allowing birds to consume only the nutritious tissues (defleshing) or partially preying on seeds^36^. Although van der Pijl^18^ classified stomatochory as a subclass within synzoochory, this may obscure its distinct ecological role as a dispersal mechanism on its own right. Notably, we observed no evidence of classical synzoochory (i.e. seed catching) during focal sampling, suggesting it plays a limited local role in our study bird community. Recent reviews confirm that stomatochory is far more widespread than synzoochory, involving a broader array of plant and bird families and species^26,28^. Epizoochory, although less frequent in our system, also challenges classical views. Traditionally considered a non-mutualistic dispersal mechanism, where seeds passively attach to animals via hooks or barbs without offering a nutritional reward^18^, recent evidence shows that it can operate mutualistically when seeds adhere to animals during feeding on viscous-pulped fruits^30,31^. This mutualistic dimension remains largely overlooked in classical dispersal syndromes and merits further research. Stomatochory is especially frequent in certain birds like parrots and corvids, and for plants bearing dry and large drupaceous fruits. Epizoochory is more common in parrots and associated with berry-like fruits^31^. While both mechanisms are still underrepresented in the literature, our observations and visual tracking suggest they facilitate seed dispersal over long distances, often hundreds of metres. In parrots, seeds have been transported up to 1620 m from the mother plant via stomatochory and epizoochory^23,25,31^. These estimates are likely conservative, as distant perches are difficult to detect, and actual dispersal distances may extend several kilometres, depending on species’ mobility^23^.

Contrary to traditional expectations, we found widespread polychory (the dispersal of seeds via multiple mechanisms) affecting nearly 50% of plant species and frequently occurring at the individual level in both plants (40%) and birds (10% of individual bird visits). In plants, endozoochory often co-occurred with other zoochories, while stomatochory was often paired with endozoochory or, less commonly, epizoochory. In our study, key dispersers, like parrots, corvids, thrushes or starlings regularly performed endozoochory combined with stomatochory. This behavioural flexibility has major ecological implications, particularly in regions where these birds are abundant, such as parrots in the tropics or corvids and starlings in temperate zones^65^. Although mixed zoochorous and/or non-zoochorous dispersal mechanisms have been reported before within plant species^11,18,23,24,27,31^, our study demonstrates that polychory is far more prevalent than previously recognised, and is still poorly understood^72^, especially compared to related processes such as diplochory (i.e. sequential dispersal modes^18^). These findings reveal that multiple interactions and dispersal mechanisms often occur simultaneously, not only at the species level but also within individuals^10,55^. Intraspecific and intra-individual variation in traits such as fruit size, for example, can attract a broader range of functionally distinct birds, enhancing the likelihood of seed dispersal. This complexity is crucial for understanding the evolutionary interplay between plants and their consumers^43,45–47^. While polychory has been documented in plants^18^, its occurrence and implications in other animals not included in our study, such as primates and bats, remain understudied. Our results emphasise the need for deeper exploration of its ecological and evolutionary consequences, particularly given its potential to reshape the structure and resilience of seed dispersal networks.

### Conclusions and future research lines

Our findings demonstrate that plant–bird interactions operate along a dynamic continuum from antagonism to mutualism and highlight the widespread occurrence of polychory. This challenges traditional views of ecosystem functioning and plant life cycles. Conventional dichotomies that categorise animals strictly as either antagonists or mutualists of plants have obscured the ambivalent functional roles many species may play, such as parrots^39,40,43,73^, true finches or pigeons^38^. Moreover, the classical emphasis on seed dispersal syndromes, together with the underestimation of birds’ capacity to overcome previously assumed morphological or behavioural constraints^54^, has perpetuated these biases. As a consequence, plant–bird interactions have often been interpreted through an overly simplified lens, leading to incomplete conclusions in systems where interaction outcomes shift along a continuum^14,15,18,39,40^, and where polychory appears to be far more widespread than previously recognised.

Our study distinguishes itself by employing detailed observations of thousands of visits by individual foraging birds and by tracking the ultimate fate of fruits and seeds. This approach has allowed us to capture the complex and dynamic processes occurring along the antagonism–mutualism continuum over short temporal scales. Although the quantitative expression of these processes may vary across communities^74,75^, we propose that similarly meticulous studies conducted in other ecological contexts would reveal comparable levels of underlying complexity. From this perspective, large-scale studies that rely primarily on trait-based approaches should be interpreted with caution when inferring global patterns of plant–animal interactions, as such approaches may lack the ecological realism required to accurately predict interactions outcomes^55^. We acknowledge that direct observation methods may be difficult to apply in certain environments, however, alternative tools, such as photo-trapping cameras offer viable alternatives^76^. Conversely, indirect methods—including DNA barcoding or analyses of seed traces in faeces—although reducing sampling effort^76^, may bias results towards endozoochory and thereby underrepresent other critical processes, such as defleshing, stomatochory or epizoochory^12,20,21^. Persisting in these methodological biases risks underestimating the ecological and evolutionary importance of stomatochory and may foster the erroneous assumption that large-fruited plants depend exclusively on specific seed disperser groups (e.g. birds on islands or extinct megafauna in the neotropics), while overlooking non-endozoochorous seed dispersal services provided by other animals, including birds, bats and rodents^23,77,78,79^. Such a narrow perspective can lead to substantial underestimations of actual dispersal distances^10,25^. Moreover, broad categorisations of species as synzoochorous, without explicit evaluation of the fate of transported fruits and seeds or consideration of local climatic conditions (e.g. tropical system where food caching can be unfavourable for seed survival^28,29^), obscure the full extent and ecological relevance of stomatochory. Our extensive fieldwork provides robust evidence that both the antagonism–mutualism continuum and the co-occurrence of multiple zoochorous mechanisms (polychory) are not only common at the species level, but also manifest within individual plants and birds. Intra-individual variability can therefore play a significant role in shaping plant–animal interactions^32,45–47^. Accordingly, intraspecific variation among seed dispersers, both morphological and behavioural, may facilitate different seed dispersal mechanisms. For instance, bold individuals may disperse seeds into more open sites than shy individuals, potentially enhancing germination success (see for review^32^). Although these approaches are more challenging to study, future research should more explicitly acknowledge their widespread prevalence in order to achieve a mechanistic understanding of plant–animal interactions and to better predict how these interactions may be altered by under current drivers of global change.

## Methods

### Study area and species

Our study was conducted in the city of Seville and its surrounding areas, in southern Spain, covering 735 km^2^ of predominantly urban and intensively cultivated landscapes (Fig. S3). The climate is mediterranean, with rainy springs and autumns and dry summers. Vegetation includes mediterranean shrubs and trees, croplands (mainly cereal and sunflower) and commercial fruit trees (mainly olive and orange). Field margins, urban parks and gardens host a variety of native and non-native ornamental trees and shrubs, offering a wide diversity of fruits. Wooded areas are mainly restricted to riparian forests along the Guadalquivir, Guadiamar and Guadaíra rivers, and some dehesas (oak woodlands) embedded within agricultural landscapes. Based on previous studies^31,65^, we selected 40 representative and abundant plant species (belonging to 18 families) regularly consumed by local avifauna (Table S2). Most selected species were arboreal or shrubby-arboreal (87.5%), with 60% being non-native and 30% cultivated (including native and non-native species) (Table S2). These species exhibited high fruit diversity, which we classified as fleshy or dry, depending on the presence or absence of a soft, juicy mesocarp (pulp). Fleshy fruits were further categorised as either drupaceous (i.e. single fruits with up to 10 seeds per fruit, including true drupes, pomes, hesperidia and some few-seeded berries) or berry-like (i.e. multiple fruits with more than 10 seeds per fruit, including berries, syconia and aggregates) (ref. ^80^; Table S2). According to the literature, most species are classified as dispersed by endozoochory (67.5%) or anemochory (20%), while the remaining 12.5% rely on myrmecochory or synzoochory. None is reported to be dispersed by stomatochory or epizoochory (Tables S2 and S3).

The diverse vegetation in our study area sustains a rich avian community, including both resident and trans-Saharan and European migrant species, mainly from the orders Passeriformes and Columbiformes. This community also includes two well-established non-native parrots (order Psittaciformes), the rose-ringed parakeet (*Psittacula krameri*) and the monk parakeet (*Myiopsitta monachus*)^81^, along with an incipient population of blue-crowned parakeet (*Thectocercus acuticaudatus*; 5–6 individuals; D. Hernández-Brito, own data). Bird feeding strategies vary widely, ranging from gulpers (which swallow fruits whole, digest the pulp, and excrete or regurgitate intact seeds) to grinders (which swallow fruits whole but digest both pulp and seeds in their muscular gizzards), crushers (which crack seeds using robust bills) and peckers (which peel pulp and peck through seed coats while holding fruits with their feet)^17^. Based on this criterion, we categorised gulpers and grinders as swallowers (species that typically ingest whole fruits), while peckers and crushers were classified as non-swallowers (birds that mainly consume fruits partially) (Table S1). Although not formally quantified, the composition, spatial distribution and relative abundance of bird species in the area are strongly influenced by human activities. This anthropogenic influence provides two key advantages for our research. First, birds are highly tolerant to human disturbance^82^, facilitating detailed behavioural observations even in challenging habitats, such as dense arboreal canopies^12,76^. Second, because most fruit-bearing plants and their avian dispersers exhibit weak, diffuse co-evolutionary relationships^5,13,28,83^, the coexistence of native and non-native plant and bird species increases morphological and behavioural diversity, broadening the spectrum of possible interactions. Notably, previous studies in the area^31,65^ and other urban communities^84^ suggest that the trophic behaviour of native and non-native birds is largely independent of any previous co-occurrence with the plant species involved.

### Field observations and data collection

Between April 2022 and May 2023, we systematically documented avian foraging behaviour across a complete annual cycle, recording interactions between birds and fruits, as well as the seed dispersal mechanisms employed. A team of 3–4 observers monitored each plant for 30 min, closely observing individual foraging birds for up to 5 min from distances of 10–50 m using optical equipment (binoculars 10 × 42 and spotting scopes 20 × 60) to minimise disturbance. Concurrently, we dedicated 120 h to locating roosts, perches, and nesting sites that were actively used by focal bird species, assessing the status of fruits and seeds dropped after their transport and consumption (Data S2 and S3). In addition, we visually examined the integrity of seeds found in faecal samples and regurgitated pellets collected from multiple bird species between 2015 and 2023 (Data S2). No ethical approval was needed as the study was observational.

Based on bird’s actions and the fate of the seeds, plant–bird interactions were classified as seed predation (complete seed destruction), fruit defleshing (pulp removal without seed damage) or seed dispersal (transport of seeds away from the mother plant by endozoochory, epizoochory, stomatochory and synzoochory). For acorns (seeds of the genus *Quercus*), partial cotyledon consumption (<60%) that preserved the embryo was functionally equivalent to pulp removal in fleshy fruits^64,85,86^, and thus classified as fruit defleshing. While most swallowed seeds were considered as dispersed by endozoochory, faecal and regurgitate analysis revealed exceptions where seeds were destroyed during digestion, namely: (1) ingestion of unripe fruits with unprotected seeds^10^, (2) ingestion of ripe fruits with small seeds (<5 mm) and soft coats (hardness 1; Table S2) by pigeons and doves^37,38^, and 3) ingestion of acorns by the wood pigeon (*Columba palumbus*), whose gizzard completely destroyed them (ref. ^87^; F. Hiraldo, own data) (Data S2). When birds carried intact seeds away from the mother plant, the interaction was classified as stomatochory (if birds actively transport fruits with intact seeds in their beaks or feet) or epizoochory (if intact seeds are passively transported attached to the bird’s bodies). In such cases, especially for multi-seeded fruits like dry or berry-like fruits, interactions were classified as seed dispersal as long as at least some intact seeds were transported, even if others were consumed. To confirm stomatochory as an effective seed dispersal mechanism, we visually tracked the birds whenever possible and examined the seeds’ fates in situ (i.e. whether they were predated, swallowed or discarded intact; Data S3). It is worth noting that unripe fruits referred to in this study were in fact close to full ripening. Although they contained chemical repellents^8,9^ or had soft seed coats (i.e. exocarp^7,11^), their seeds were capable of germinating after separation from the mother plant^11,60,86^. Rare cases of birds consuming genuinely immature seeds—those whose embryos could not survive detachment were excluded from the analyses due to low sample sizes (between 2.2 and 19.3% of total consumed fruits from five plant species exclusively consumed by three bird species) (Table S11). Synzoochory, defined as the deliberate external transport and subsequent hoarding of seeds^18^, was too rarely observed to be analysed (*n* = 6 events of Eurasian magpies *Pica pica* hoarding acorns; Data S3).

Although our interaction classification was based on seed fate, we used fruits as the primary sampling unit for quantitative analysis. This decision reflects the inherent difficulties in tracking individual seeds and the substantial variation in seed size and number. Fruit-based quantification, a standard approach in similar ecological studies, offers a consistent and comparable metric, even though it precludes exact measurement of the relative contribution of each interaction or dispersal mechanism^19^. Nevertheless, given field limitations, it represents the most practical and reliable strategy^22,25,29,64^.

### Statistical analyses

We implemented Bayesian (generalised) linear mixed models using the *brms* package^88^ to investigate the factors influencing whether birds consume fruit pulp (defleshing), predate seeds (predation) or dispersed them (dispersal) and, for dispersal events, the probability of doing so via endozoochory, stomatochory or epizoochory. Fixed effects included fruit type (dry, drupaceous or berry-like), ripeness (unripe or ripe), feeding strategy (swallowers and non-swallowers), and the fruit-to-bird size ratio, calculated from fruit length (mm) and bird body mass (g) (Tables S1 and S2). We tested both additive and interactive effects, incorporating linear and quadratic terms for the continuous variable (fruit-to-bird size ratio) to capture potential non-linear relationships. Ripeness was excluded from seed dispersal models because seeds dispersed at both ripeness stages were capable of germination^60^. To account for shared evolutionary history among closely related species, we incorporated plant and bird phylogenies derived from the Open Tree of Life synthetic tree^89^ and BirdTree.org^90^ using the *rotl* package^91^. We also included plant and bird species as random terms to control for pseudoreplication and quantify the extent to which species identity explains variation in interaction outcomes and dispersal mechanisms, independently of phylogenetic relationships.

We further investigated the potential drivers of intraspecific variation by examining whether it stems from individual flexibility (i.e. single individuals exhibiting multiple roles and/or dispersal mechanisms) or from the coexistence of individuals that specialise in distinct roles or dispersal mechanisms, collectively broadening the species’ ecological spectrum. To address this, we used the same modelling framework to assess the probability that individuals simultaneously engage in multiple interaction types and seed dispersal strategies, incorporating fruit type, ripeness, and the fruit-bird size ratio as explanatory variables. It should be noted that our individual bird observations were conducted on unmarked individuals; therefore, they do not represent strictly identified individuals. While focal birds were monitored while they remained within the focal plant or its immediate surroundings after foraging visits—thus partially controlling for pseudoreplication—we could not ascertain individual identity once birds left the observation area and subsequently returned. Despite this limitation, our assessment at the individual level remains valid, as the models capture the variability in interactions and dispersal mechanisms within the observation windows. This approach ensures that our estimates reflect short-term behavioural variability relevant to ecological interactions, even if exact individual identity cannot be guaranteed. For plant-level models, the fruit-to-bird-size ratio was averaged across all birds recorded per plant. Bird-level models also incorporated the duration of each individual foraging bout (maximum 5 min), while plant-level models included the abundance of foraging birds, their taxonomic diversity (measured using the Shannon index), cumulative foraging time and the proportion of birds consuming unripe and ripe fruits during the 30-min monitoring session as a proxy for resource diversity.

For both plant–bird interaction and seed dispersal models, models were built by first fitting the highest-order interaction (4-way interaction for plant–bird interaction models and 3-way interaction for dispersal models). Significance of interaction terms and main effects was assessed by checking whether the 95% credible interval (CI) of the posterior distribution overlapped zero, and non-significant terms were removed stepwise to identify the most parsimonious model. For the individual-level models, we applied the same procedure, retaining all contextual covariates (i.e. foraging time, consumer diversity and consumer abundance) to control for their effects. From the final models’ (co)variance matrices, we estimated phylogenetic signal (*λ* and 95% CIs) for each interaction type and dispersal mechanism at both the fruit and the individual bird visit and plant. We also calculated the variance explained by all parameters (conditional *R*^2^) and by fixed effects alone (marginal *R*^2^) through the *rstantools* package^92^, and we decomposed the variance components for all random effects in the final models using the *varde* package^93^. We performed post hoc tests using the *emmeans* package^94^ to estimate the marginal means for each pairwise combination of categorical explanatory variables (fruit type, ripeness and feeding strategy).

All models used a binomial (Bernoulli) error distribution with a logit link function. We specified normally distributed priors, weakly informative for fixed and random effects (mean = 0, SD = 2) and moderated-informative for the intercept (mean = −3, SD = 2) to better model rare events. Models were run over four independent chains with 3500 iterations, with a warmup/burn-in of 1750 to ensure a minimum effective sample size (bulk-ESS and tail-ESS) above 400 for all parameters (random and fixed effects). Convergence was confirmed using Rhat values ≤ 1.01.

For visualisation of continuous fixed effects, we extracted the values predicted by the models and re-estimated them using the 'geom_smooth' function (generalised linear models, binomial error distribution, logit link function), with shaded areas indicating the 95% confidence interval (Figs. 2, 5 and 6). For visualisation of categorical fixed effects, we used bar plots portraying the mean predicted values, with whiskers denoting 95% confidence intervals (Fig. 6). To visualise the percentages of recorded plant–bird interactions and seed dispersal mechanisms across the branches of both phylogenies (Figs. 3 and 4), we used the 'contMap' function from the *phytools* package^95^. Finally, to illustrate the dynamic nature of interactions and dispersal mechanisms, we calculated transition probabilities from seed predation to the various dispersal modes for each fruit type (Table S5). All analyses were conducted in R 4.0.3^96^.

### Reporting summary

Further information on research design is available in the Nature Portfolio Reporting Summary linked to this article.

## Supplementary information

Supplementary Information

Description of Additional Supplementary Files

Supplementary Data 1

Supplementary Data 2

Supplementary Data 3

Reporting Summary

## Data Availability

All the data supporting this work are available from the main article and the Supplementary Information.
