# Supplementary material for: Context-dependent plant–bird interactions shape polychory across the antagonism–mutualism continuum
Source: Commun Biol. 2026 Apr 28;9:739. doi: 10.1038/s42003-026-10142-x (PMC13226708; doi:10.1038/s42003-026-10142-x)
Supplement: Supplementary file 1 — Supplementary Information [file 42003_2026_10142_MOESM1_ESM.pdf]

SUPPLEMENTARY MATERIAL

Context-dependent plant–bird interactions shape polychory across the antagonism–mutualism continuum

Dailos Hernández-Brito, Fernando Hiraldo, Jaume Izquierdo-Palma, José L. Tella and Martina Carrete

[1] Department of Conservation Biology, Estación Biológica de Doñana (CSIC), Seville, Spain

[2] Department of Physical, Chemical and Natural Systems, Universidad Pablo de Olavide, Seville, Spain

*dailoshb@gmail.com (correspondence author)

Table S1. Body mass (in g) and feeding strategies (i.e., swallower and non-swallower) of bird species observed feeding on fruits and seeds during the study. Body masses were obtained from Dunning (2007)[1] and feeding strategies were categorized following Yoshikawa et al. (2009)[2]. Swallowers include species that ingest fruits whole, either gulpers (i.e., which digest the pulp and excrete or regurgitate intact seeds) or grinders (i.e., which digest both pulp and seeds in their muscular gizzards). Non-swallowers are birds that consume fruits partially, either crushers (i.e., which crack seeds using strong bills) or peckers (i.e., which peel the pulp and pierce seed coats while holding fruits with their feet). Non-native species present in the study area are indicated with an asterisk. Geographic ranges were obtained from Billerman et al. (2025)[3].

Order	Family	Bird species	Body mass (g)	Feeding strategy	Geographic range
**Columbiformes**	Columbidae	*Columba livia*	354.5	Swallower (Grinder)	Macaronesia-Eurasia-N Africa-Sahel
		Columba palumbus	490	Swallower (Grinder)	Europe / Near East
		Streptopelia decaocto	149	Swallower (Grinder)	Macaronesia / Eurasia / NW Africa
**Passeriformes**	Corvidae	*Coloeus monedula*	246	Swallower (Gulper)	Europe / NW Asia
		Cyanopica cooki	96	Swallower (Gulper)	Iberian Peninsula
		Pica pica	206	Swallower (Gulper)	Eurasia / NW Africa
	Fringillidae	*Carduelis carduelis*	16	Non-swallower (Crusher)	Macaronesia / Europe / W Asia / Irano-Turanian
		Chloris chloris	26	Non-swallower (Crusher)	Macaronesia / Europe / NW Asia
		Fringilla coelebs	21.4	Non-swallower (Crusher)	Europe / NW Asia
		Linaria cannabina	19.55	Non-swallower (Crusher)	Macaronesia / Europe / N Africa / NW Asia / Irano-Turanian
		Serinus serinus	11.2	Non-swallower (Crusher)	Europe / Mediterranean
	Oriolidae	*Oriolus oriolus*	79	Swallower (Gulper)	Europe / NW Asia / C-SE Africa
	Passeridae	*Passer domesticus*	27.7	Non-swallower (Crusher)	Europe / N Africa / NW Asia / Indian subcontinent
		Passer hispaniolensis	34.2	Non-swallower (Crusher)	Macaronesia / Mediterranean / Middle East / NW India

		Passer montanus	22	Non-swallower (Crusher)	Europe / W-SE Asia
	Sturnidae	*Sturnus unicolor*	85	Swallower (Gulper)	W Mediterranean
	Sylviidae	*Curruca communis*	15.1	Swallower (Gulper)	Europe / NW Asia / S Sahel / CW Africa
		Curruca melanocephala	11.7	Swallower (Gulper)	Macaronesia / Mediterranean / Sahel
		Sylvia atricapilla	16.7	Swallower (Gulper)	Macaronesia / Europe / N Africa / NW Asia / Ecuatorial Africa
		Sylvia borin	18.2	Swallower (Gulper)	Europa / NW Asia / Sub-Saharan Africa
	Turdidae	*Turdus merula*	113	Swallower (Gulper)	Europe / N Africa / Middle East / W China
		Turdus philomelos	67.75	Swallower (Gulper)	Europa / N Africa / NW Asia
**Psittaciformes**	Psittacidae	*Myiopsitta monachus**	120	Non-swallower (Peckers)	S Bolivia / N Southern Cone
		*Thectocercus acuticaudatus**	171	Non-swallower (Peckers)	Caribbean South America / N Southern Cone
	Psittaculidae	*Psittacula krameri**	126	Non-swallower (Peckers)	Indian subcontinent / S Sahel

[1]Dunning, Jr, J. B. (2007). CRC handbook of avian body masses. Boca Raton, FL: CRC press.

[2]Yoshikawa, T., Isagi, Y. and Kikuzawa, K. (2009), Relationships between bird-dispersed plants and avian fruit consumers with different feeding strategies in Japan. Ecol. Res., 24: 1301-1311 1301.

[3]Billerman, S. M., B. K. Keeney, G. M. Kirwan, F. Medrano, N. D. Sly, and M. G. Smith, Editors (2025). Birds of the World. Cornell Laboratory of Ornithology, Ithaca, NY, USA. https://birdsoftheworld.org/bow/home

Table S2. Fruit characteristics of the study plant species. Fruits were classified (Fruit type) as fleshy or dry, based on the presence or absence of a fleshy mesocarp (pulp) and, within fleshy fruits and according to the number of seeds per fruit, as drupaceous (i.e., single fruits with up to 10 seeds per fruit, including true drupes, pomes, hesperidia, and some few-seeded berries) or berry-like (i.e., multiple fruits with more than 10 seeds per fruit, including berries, syconia, and aggregates) [4]. As there is no standard measure available for all the studied plants, seed hardness (Hardness) was categorized into three levels representing increasing bite force required to deform or compress a seed. These levels were based on manual testing of 20 seeds per species (including equal numbers of mature and immature seeds; mature/immature), conducted by the same person (DHB)[5]. Geographic ranges were derived from Castroviejo (2012)[6] and POWO (2024)[7]. Fruit size (measured as the larger of fruit length or width, in mm), growth habit, seed dispersal syndrome and mean number of seeds per fruit were obtained from the literature (Table S3). Non-native species are marked with an asterisk (*) and cultivated species with a plus sign (+).

Plant species	Family	Fruit type	Growth habit	Seed dispersal syndrome	Geographic range	Fruit size (mm)	Seed hardness	Seeds per fruit (mean)
Arbutus unedo	Ericaceae	Berry-like	Shrub-tree	Endozoochory	Macaronesia / Ireland/ S Europe / Mediterranean	17.1	1/1	12.9
*Campsis radicans**	Bignoniaceae	Dry	Liana	Anemochory	E North America	160	1/1	696
*Catalpa bignonioides**	Bignoniaceae	Dry	Tree	Anemochory	SE USA	300	1/1	94.1
Celtis australis	Ulmaceae	Drupaceous	Tree	Endozoochory	S Europe / W Asia / N Africa	10.1	3/2	1
Ceratonia siliqua+	Leguminosae	Dry	Shrub-tree	Endozoochory	Mediterranean	200	3/1	10
*Cercis siliquastrum**	Leguminosae	Dry	Shrub-tree	Endozoochory	SE Europe / Middle East	75	3/1	7
*Citrus ˣ aurantium**+	Rutaceae	Berry-like	Shrub-tree	Endozoochory	China	80	2/1	17.82
*Cupressus sempervirens**	Cupressaceae	Dry	Tree	Anemochory	Aegean	32	2/2	152.08
*Eriobotrya japonica**+	Rosaceae	Drupaceous	Shrub-tree	Endozoochory	W China / Japan	41.1	1/1	1.5
*Feijoa sellowiana**+	Myrtaceae	Berry-like	Shrub-tree	Endozoochory	N Southern Cone	74.7	1/1	80.33

Ficus carica+	Moraceae	Berry-like	Tree	Endozoochory / Myrmecochory	Mediterranean / Middle East / Pakistan	33.5	1/1	1215
*Ficus microcarpa**	Moraceae	Berry-like	Tree	Endozoochory / Myrmecochory	Indian subcontinent / SE Asia	8	1/1	168
Fraxinus angustifolia	Oleaceae	Dry	Tree	Anemochory	S Europe /SW Asia / NW Africa	28	1/1	1
Fraxinus excelsior	Oleaceae	Dry	Tree	Anemochory	S Europe / W Asia	36.5	1/1	1
*Helianthus annuus**+	Compositae	Dry	Herb	Myrmecochory / Dyszoochory	NW North America	188.3	1/1	591.63
*Lantana ˣ strigocamara**	Verbenaceae	Drupaceous	Shrub	Endozoochory	Caribbean / Central / America / N South America	5.5	2/1	1
*Ligustrum japonicum**	Oleaceae	Drupaceous	Shrub-tree	Endozoochory	Korea / Japan	4.2	1/1	1
*Livistona chinensis**	Arecaceae	Drupaceous	Tree	Endozoochory	SE China / Japan	30	3/2	1
*Magnolia grandiflora**	Magnoliaceae	Dry	Tree	Endozoochory	SE USA	68.7	1/1	64.18
*Melia azedarach**	Meliaceae	Drupaceous	Tree	Endozoochory	Indian subcontinent / SE Asia / Japan / N Australia	16	3/2	3.61
*Morus alba**+	Moraceae	Berry-like	Tree	Endozoochory	CE China / Korea	19.5	1/1	17.8
*Morus nigra**+	Moraceae	Berry-like	Tree	Endozoochory	Anatolia	24.5	1/1	18
Myrtus communis	Myrtaceae	Berry-like	Shrub-tree	Endozoochory / Myrmecochory	Mediterranean	8.5	1/1	5.2
Olea europaea+	Oleaceae	Drupaceous	Shrub-tree	Endozoochory	Mediterranean	25.3	3/2	1
Olea europaea europaea	Oleaceae	Drupaceous	Shrub-tree	Endozoochory	Mediterranean	11.3	3/2	1
*Phoenix canariensis**	Arecaceae	Drupaceous	Tree	Endozoochory	Canary Islands	20.2	3/2	1
*Phoenix dactylifera**+	Arecaceae	Drupaceous	Tree	Endozoochory	NE Sahara / Middle East	46.2	3/2	1
Pistacia lentiscus	Anacardiaceae	Drupaceous	Shrub-tree	Endozoochory / Myrmecochory	Canary Islands / Mediterranean	5.3	2/1	1
*Platycladus orientalis**	Cupressaceae	Dry	Shrub-tree	Anemochory	China	21.3	2/1	251.5
*Prunus dulcis**+	Rosaceae	Dry	Tree	Endozoochory / Synzoochory	Balkans / SW Asia / N Africa	36.2	3/3	1
*Punica granatum**+	Lythraceae	Berry-like	Tree	Endozoochory	Irano-Turanian	81.9	2/1	384.09
Pyrus bourgaeana	Rosaceae	Drupaceous	Tree	Endozoochory	Iberian Peninsula / NW Africa	25	1/1	7.9
Quercus ilex	Fagaceae	Dry	Shrub-tree	Synzoochory	Mediterranean	36.2	1/1	1
Quercus suber	Fagaceae	Dry	Tree	Synzoochory	W Mediterranean	30.5	1/1	1

Rubus ulmifolius	Rosaceae	Berry-like	Subshrub	Endozoochory	Macaronesia / W Europe / NW Africa	13.1	1/1	28.8
Silybum marianum	Compositae	Dry	Herb	Myrmecochory	Mediterranean / SW Asia / Indian subcontinent	48	2/1	150
*Styphnolobium japonicum**	Leguminosae	Drupaceous	Tree	Endozoochory	E China	75	2/1	3.5
*Tipuana tipu**	Leguminosae	Dry	Tree	Anemochory	Bolivia / N Southern Cone	55	3/2	1.4
Ulmus minor	Ulmaceae	Dry	Tree	Anemochory	Europe / NW Asia	15	1/1	1
*Washingtonia robusta**	Arecaceae	Drupaceous	Tree	Endozoochory	SW USA / N Mexico	9.2	3/2	1

[4]Guimarães Jr, P.R., Galetti, M. & Jordano, P. (2008). Seed dispersal anachronisms: rethinking the fruits extinct megafauna ate. PloS one, 3(3), e1745.

[5]Alcaraz-Mármol, F., Calín-Sánchez, Á., Nuncio-Jáuregui, N., Carbonell-Barrachina, Á. A., Hernández, F., & Martínez, J. J. (2015). Classification of pomegranate cultivars according to their seed hardness and wood perception. Journal of Texture Studies, 46(6), 467-474.

[6]Castroviejo, S. (coord. gen.). 1986-2012. Flora iberica 1-8, 10-15, 17-18, 21. Real Jardín Botánico, CSIC, Madrid.

[7]POWO (2024). "Plants of the World Online. Facilitated by the Royal Botanic Gardens, Kew. Published on the Internet; https://powo.science.kew.org/

Table S3. Bibliographic sources used to obtain plant trait data (see Table S2).

Arbutus unedo
Jordano, P. (1995). Angiosperm fleshy fruits and seed dispersers: a comparative analysis of adaptation and constraints in plant-animal interactions. The American Naturalist, 145(2), 163-191. https://doi.org/10.1086/285735
Lososová, Z., Axmanová, I., Chytrý, M., Midolo, G., Abdulhak, S., Karger, D. N., ... & Thuiller, W. (2023). Seed dispersal distance classes and dispersal modes for the European flora. Global Ecology and Biogeography, 32(9), 1485-1494. https://doi.org/10.1111/geb.13712
Campsis radicans
Chachalis, D., & Reddy, K. N. (2000). Factors affecting Campsis radicans seed germination and seedling emergence. Weed science, 48(2), 212-216. http://dx.doi.org/10.1614/0043-1745(2000)048[0212:FACRSG]2.0.CO;2
Lososová, Z., Axmanová, I., Chytrý, M., Midolo, G., Abdulhak, S., Karger, D. N., ... & Thuiller, W. (2023). Seed dispersal distance classes and dispersal modes for the European flora. Global Ecology and Biogeography, 32(9), 1485-1494. https://doi.org/10.1111/geb.13712
Catalpa bignonioides
Olsen, R. T., & Kirkbride Jr, J. H. (2017). Taxonomic revision of the genus Catalpa (Bignoniaceae). Brittonia, 69(3), 387-421. https://doi.org/10.1007/s12228-017-9471-7
Olsen, R. T. (2007). Utilizing Polyploidy for Developing Improved Nursery Crops: Restoring Fertility in Wide Hybrids, Limiting Fertility of Invasive Species, Embryo Culture of Triploids, Pest Resistance, and Inheritance of Ornamental Traits. http://www.lib.ncsu.edu/resolver/1840.16/3495
Lososová, Z., Axmanová, I., Chytrý, M., Midolo, G., Abdulhak, S., Karger, D. N., ... & Thuiller, W. (2023). Seed dispersal distance classes and dispersal modes for the European flora. Global Ecology and Biogeography, 32(9), 1485-1494. https://doi.org/10.1111/geb.13712

Celtis australis
Jordano, P. (1995). Angiosperm fleshy fruits and seed dispersers: a comparative analysis of adaptation and constraints in plant-animal interactions. The American Naturalist, 145(2), 163-191. https://doi.org/10.1086/285735<br><br>Lososová, Z., Axmanová, I., Chytrý, M., Midolo, G., Abdulhak, S., Karger, D. N., ... & Thuiller, W. (2023). Seed dispersal distance classes and dispersal modes for the European flora. Global Ecology and Biogeography, 32(9), 1485-1494. https://doi.org/10.1111/geb.13712
Ceratonia siliqua
Batlle, I. and J. Tous. 1997. Carob tree. Ceratonia siliqua L. Promoting the conservation and use of underutilized and neglected crops. 17. Institute of Plant Genetics and Crop Research, Gatersleben/International Plant Genetic Resources Institute, Rome, Italy. Plant<br><br>Sidina, M. M., El Hansali, M., Wahid, N., Ouatmane, A., Boulli, A., & Haddioui, A. (2009). Fruit and seed diversity of domesticated carob (Ceratonia siliqua L.) in Morocco. Scientia horticulturae, 123(1), 110-116. https://doi.org/10.1016/j.scienta.2009.07.009<br><br>Lososová, Z., Axmanová, I., Chytrý, M., Midolo, G., Abdulhak, S., Karger, D. N., ... & Thuiller, W. (2023). Seed dispersal distance classes and dispersal modes for the European flora. Global Ecology and Biogeography, 32(9), 1485-1494. https://doi.org/10.1111/geb.13712
Cercis siliquastrum
Dickson, J. G. (1990). Cercis canadensis L. Eastern redbud. Silvics of North America, 2, 266-269. https://www.srs.fs.usda.gov/pubs/misc/ag_654/volume_2/cercis/canadensis.htm<br><br>Lososová, Z., Axmanová, I., Chytrý, M., Midolo, G., Abdulhak, S., Karger, D. N., ... & Thuiller, W. (2023). Seed dispersal distance classes and dispersal modes for the

European flora. Global Ecology and Biogeography, 32(9), 1485-1494. https://doi.org/10.1111/geb.13712

Citrus ˣ aurantium

Etebu, E., & Nwauzoma, A. B. (2014). A review on sweet orange (Citrus sinensis L Osbeck): health, diseases and management. American Journal of Research Communication, 2(2), 33-70. http://www.usa-journals.com/wp-content/uploads/2014/01/Etebu_Vol22.pdf

Anand, J., Rawat, J. S., Rawat, V., Singh, B., Khanduri, V. P., Riyal, M. K., ... & Kumar, M. (2022). Climatic and Altitudinal Variation in Physicochemical Properties of Citrus sinensis in India. Land, 11(11), 2033. https://doi.org/10.3390/land11112033

Lososová, Z., Axmanová, I., Chytrý, M., Midolo, G., Abdulhak, S., Karger, D. N., ... & Thuiller, W. (2023). Seed dispersal distance classes and dispersal modes for the European flora. Global Ecology and Biogeography, 32(9), 1485-1494. https://doi.org/10.1111/geb.13712

Cupressus sempervirens

Al-Snafi, A. E. (2016). Medical importance of Cupressus sempervirens-A review. IOSR Journal of Pharmacy, 6(6), 66-76.

Sękiewicz, K., Boratyńska, K., Dagher-Kharrat, M. B., Ok, T., & Boratyński, A. (2016). Taxonomic differentiation of Cupressus sempervirens and C. atlantica based on morphometric evidence. Systematics and Biodiversity, 14(5), 494-508. https://doi.org/10.1080/14772000.2016.1171260

Lososová, Z., Axmanová, I., Chytrý, M., Midolo, G., Abdulhak, S., Karger, D. N., ... & Thuiller, W. (2023). Seed dispersal distance classes and dispersal modes for the European flora. Global Ecology and Biogeography, 32(9), 1485-1494. https://doi.org/10.1111/geb.13712

Eriobotrya japonica

Hussain, A., 2009. Study of seasonal biomass productivity and nutritional quality of major forage species in subtropical sub humid rangelands of district Chakwal. PhD Thesis, Pir Mehr Ali Shah Arid Agriculture University, Rawalpindi, 379 p.<br><br>Lososová, Z., Axmanová, I., Chytrý, M., Midolo, G., Abdulhak, S., Karger, D. N., ... & Thuiller, W. (2023). Seed dispersal distance classes and dispersal modes for the European flora. Global Ecology and Biogeography, 32(9), 1485-1494. https://doi.org/10.1111/geb.13712
Feijoa sellowiana
Patterson, K. J. (1990). Effects of pollination on fruit set, size, and quality in feijoa (Acca sellowiana (Berg) Burret). New Zealand Journal of Crop and Horticultural Science, 18(2-3), 127-131. https://doi.org/10.1080/01140671.1990.10428082<br><br>Gressler, Eliana, Marco A. Pizo, and L. Patrícia C. Morellato. "Polinização e dispersão de sementes em Myrtaceae do Brasil." Brazilian Journal of Botany 29 (2006): 509-530. https://doi.org/10.1590/S0100-84042006000400002
Ficus carica
Sebastián-González, E., Hiraldo, F., Blanco, G., Hernandez-Brito, D., Romero-Vidal, P., Carrete, M., ... & Tella, J. L. (2019). The extent, frequency and ecological functions of food wasting by parrots. Scientific Reports, 9(1), 15280. https://doi.org/10.1038/s41598-019-51430-3<br><br>Lososová, Z., Axmanová, I., Chytrý, M., Midolo, G., Abdulhak, S., Karger, D. N., ... & Thuiller, W. (2023). Seed dispersal distance classes and dispersal modes for the European flora. Global Ecology and Biogeography, 32(9), 1485-1494. https://doi.org/10.1111/geb.13712<br><br>Bain, A., Harrison, R. D., & Schatz, B. (2014). How to be an ant on figs. Acta Oecologica, 57, 97-108. https://doi.org/10.1016/j.actao.2013.05.006
Ficus microcarpa

Starr, F., Starr, K., & Loope, L. (2003). Ficus microcarpa. Chinese Banyan, Moraceae. United States Geological Survey-Biological Resources Division Haleakala Field Station, Maui, Hawai'i.

Mathew et al. 2011: Mathew, G., Skaria, B. P., & Joseph, A. (2011). Standardization of conventional propagation techniques for four medicinal species of genus Ficus Linn. https://nopr.niscpr.res.in/handle/123456789/11546

Kaufmann, S., McKey, D. B., Hossaert-McKey, M., & Horvitz, C. C. (1991). Adaptations for a two-phase seed dispersal system involving vertebrates and ants in a hemiepiphytic fig (Ficus microcarpa: Moraceae). American Journal of Botany, 78(7), 971-977. https://doi.org/10.1002/j.1537-2197.1991.tb14501.x

Fraxinus angustifolia

Kaveh, M., Tavassoli, A., Azadi, R., & Memariani, F. (2014). Morphology and micromorphology of the genus Fraxinus L. in Iran. The Iranian Journal of Botany, 20(2), 188-200. https://doi.org/10.22092/ijb.2014.11020

Lososová, Z., Axmanová, I., Chytrý, M., Midolo, G., Abdulhak, S., Karger, D. N., ... & Thuiller, W. (2023). Seed dispersal distance classes and dispersal modes for the European flora. Global Ecology and Biogeography, 32(9), 1485-1494. https://doi.org/10.1111/geb.13712

Fraxinus excelsior

Kaveh, M., Tavassoli, A., Azadi, R., & Memariani, F. (2014). Morphology and micromorphology of the genus Fraxinus L. in Iran. The Iranian Journal of Botany, 20(2), 188-200. https://doi.org/10.22092/ijb.2014.11020

Lososová, Z., Axmanová, I., Chytrý, M., Midolo, G., Abdulhak, S., Karger, D. N., ... & Thuiller, W. (2023). Seed dispersal distance classes and dispersal modes for the European flora. Global Ecology and Biogeography, 32(9), 1485-1494. https://doi.org/10.1111/geb.13712

Helianthus annuus

Göksoy, A. T., Türkeç, A., & Turan, Z. M. (1999). A Research on the Analysis of Heterotic Effects for Certain Agronomical Characters in Cross Population of Sunflower (Helianthus annuus L.). Turkish Journal of Agriculture and Forestry, 23(7), 247-256. https://journals.tubitak.gov.tr/agriculture/vol23/iss7/29/

Heiser, C.B. (1976). The sunflower. University of Oklahoma Press, Norman. https://id.oclc.org/worldcat/entity/E39PBJyxcWXTrvbYJCD7gfyGHC / https://www.biodiversitylibrary.org/item/279447#page/1/mode/1up

Alcolea, M., Durigan, G., & Christianini, A. V. (2022). Prescribed fire enhances seed removal by ants in a Neotropical savanna. Biotropica, 54(1), 125-134. https://doi.org/10.1111/btp.13036

Lantana ˣ strigocamara

Ramaswami, G., Somnath, P., & Quader, S. (2017). Plant-disperser mutualisms in a semi-arid habitat invaded by Lantana camara L. Plant Ecology, 218, 935-946. https://doi.org/10.1007/s11258-017-0741-y

Barbosa, Karina C., and Marco A. Pizo. "Seed rain and seed limitation in a planted gallery forest in Brazil." Restoration Ecology 14.4 (2006): 504-515. https://doi.org/10.1111/j.1526-100X.2006.00162.x

Ligustrum japonicum

Xolmurotov, M. (2023). Fruit and seed morphology of Japanese ligustrum (Ligustrum japonicum) and Chinese ligustrum (Ligustrum sinense). Journal of Agriculture & Horticulture, 3(5), 61-64.

Lososová, Z., Axmanová, I., Chytrý, M., Midolo, G., Abdulhak, S., Karger, D. N., ... & Thuiller, W. (2023). Seed dispersal distance classes and dispersal modes for the European flora. Global Ecology and Biogeography, 32(9), 1485-1494. https://doi.org/10.1111/geb.13712

Livistona chinensis

Sebastián-González, E., Hiraldo, F., Blanco, G., Hernandez-Brito, D., Romero-Vidal, P., Carrete, M., ... & Tella, J. L. (2019). The extent, frequency and ecological

functions of food wasting by parrots. Scientific Reports, 9(1), 15280. https://doi.org/10.1038/s41598-019-51430-3

Lososová, Z., Axmanová, I., Chytrý, M., Midolo, G., Abdulhak, S., Karger, D. N., ... & Thuiller, W. (2023). Seed dispersal distance classes and dispersal modes for the European flora. Global Ecology and Biogeography, 32(9), 1485-1494. https://doi.org/10.1111/geb.13712

Magnolia grandiflora

Liu, Y., Ma, Q., Liu, H., Zhu, Y., & Zhu, Y. (2014). Phenotypic diversity of fruits and seeds of Magnolia grandiflora superior trees. Acta Agriculturae Shanghai, 30(2), 65-67. https://www.nyxb.sh.cn/CN/abstract/abstract2853.shtml

Lovisetto, A., Masiero, S., Rahim, M. A., Mendes, M. A. M., & Casadoro, G. (2015). Fleshy seeds form in the basal angiosperm Magnolia grandiflora and several MADS-box genes are expressed as fleshy seed tissues develop. Evolution & Development, 17(1), 82-91. https://doi.org/10.1111/ede.12106

Melia azedarach

Jordano, P. (1995). Angiosperm fleshy fruits and seed dispersers: a comparative analysis of adaptation and constraints in plant-animal interactions. The American Naturalist, 145(2), 163-191. https://doi.org/10.1086/285735

Chen, L., Deng, X., Ding, M., Liu, M., Li, J., Hui, W., ... & Chen, X. (2014). Geographic variation in traits of fruit stones and seeds of Melia azedarach. Journal of Beijing Forestry University, 36(1), 15-20. http://j.bjfu.edu.cn/en/article/id/9954

Lososová, Z., Axmanová, I., Chytrý, M., Midolo, G., Abdulhak, S., Karger, D. N., ... & Thuiller, W. (2023). Seed dispersal distance classes and dispersal modes for the European flora. Global Ecology and Biogeography, 32(9), 1485-1494. https://doi.org/10.1111/geb.13712

Morus alba

Hashemi, S., & Khadivi, A. (2020). Morphological and pomological characteristics of white mulberry (Morus alba L.) accessions. Scientia Horticulturae, 259, 108827. https://doi.org/10.1016/j.scienta.2019.108827

Aydın, E., Bostan, S. Z., Şen, S. M., Yarılgaç, T., Er, E., Aksu Uslu, N., & Turan, A. (2015, September). Selection of mulberry (Morus alba) in Artvin. In Third Balkan Symposium on Fruit Growing (pp. 16-18). https://doi.org/10.17660/ActaHortic.2016.1139.5

Lososová, Z., Axmanová, I., Chytrý, M., Midolo, G., Abdulhak, S., Karger, D. N., ... & Thuiller, W. (2023). Seed dispersal distance classes and dispersal modes for the European flora. Global Ecology and Biogeography, 32(9), 1485-1494. https://doi.org/10.1111/geb.13712

Morus nigra

Hosseini, A. S., Akramian, M., Khadivi, A., & Salehi-Arjmand, H. (2018). Phenotypic and chemical variation of black mulberry (Morus nigra) genotypes. Industrial Crops and Products, 117, 260-271. https://doi.org/10.1016/j.indcrop.2018.03.007

Gunes, M. E. H. M. E. T., & Cekic, C. (2004). Some chemical and physical properties of fruits of different mulberry species commonly grown in Anatolia, Turkey. Asian J. Chem, 16(3), 1849-1855.

Lososová, Z., Axmanová, I., Chytrý, M., Midolo, G., Abdulhak, S., Karger, D. N., ... & Thuiller, W. (2023). Seed dispersal distance classes and dispersal modes for the European flora. Global Ecology and Biogeography, 32(9), 1485-1494. https://doi.org/10.1111/geb.13712

Myrtus communis

Monge, R. A., & Martínez, M. F. (2012). Myrtus communis L. https://www.researchgate.net/profile/Reyes_Alejano/publication/257941420_Myrtus_communis_L/links/02e7e526659bc3116b000000.pdf

Lososová, Z., Axmanová, I., Chytrý, M., Midolo, G., Abdulhak, S., Karger, D. N., ... & Thuiller, W. (2023). Seed dispersal distance classes and dispersal modes for the

European flora. Global Ecology and Biogeography, 32(9), 1485-1494. https://doi.org/10.1111/geb.13712

Ciccarelli, D., Andreucci, A. C., Pagni, A. M., & Garbari, F. (2005). Structure and development of the elaiosome in Myrtus communis L.(Myrtaceae) seeds. Flora-Morphology, Distribution, Functional Ecology of Plants, 200(4), 326-331. https://doi.org/10.1016/j.flora.2004.12.004

Olea europaea

Kılıçkan, A., & Güner, M. E. T. İ. N. (2008). Physical properties and mechanical behavior of olive fruits (Olea europaea L.) under compression loading. Journal of Food Engineering, 87(2), 222-228. https://doi.org/10.1016/j.jfoodeng.2007.11.028

Lososová, Z., Axmanová, I., Chytrý, M., Midolo, G., Abdulhak, S., Karger, D. N., ... & Thuiller, W. (2023). Seed dispersal distance classes and dispersal modes for the European flora. Global Ecology and Biogeography, 32(9), 1485-1494. https://doi.org/10.1111/geb.13712

Olea europaea europaea

Mulas, M. (1999. Characterisation of olive wild ecotypes. In III International Symposium on Olive Growing 474 (pp. 121-124). Acta Horticulturae, (474), 121–124. https://doi.org/10.17660/ActaHortic.1999.474.21

Lososová, Z., Axmanová, I., Chytrý, M., Midolo, G., Abdulhak, S., Karger, D. N., ... & Thuiller, W. (2023). Seed dispersal distance classes and dispersal modes for the European flora. Global Ecology and Biogeography, 32(9), 1485-1494. https://doi.org/10.1111/geb.13712

Phoenix canariensis

Spennemann, D. H., Pike, M., & Robinson, W. (2020). Germination rates of old and fresh seeds and their implications on invasiveness of the ornamental Canary Islands date palm (Phoenix canariensis). European Journal of Ecology, 6(2). https://doi.org/10.17161/eurojecol.v6i2.13474

Lososová, Z., Axmanová, I., Chytrý, M., Midolo, G., Abdulhak, S., Karger, D. N., ... & Thuiller, W. (2023). Seed dispersal distance classes and dispersal modes for the European flora. Global Ecology and Biogeography, 32(9), 1485-1494. https://doi.org/10.1111/geb.13712

Phoenix dactylifera

Simozrag et al. 2016: Simozrag, A., Chala, A., Djerouni, A., & Bentchikou, M. E. (2016). Phenotypic diversity of date palm cultivars (Phoenix dactylifera L.) from Algeria. Gayana Botanica, 73(1), 42-53. https://revistas.udec.cl/index.php/gayana_botanica/article/view/4048

Lososová, Z., Axmanová, I., Chytrý, M., Midolo, G., Abdulhak, S., Karger, D. N., ... & Thuiller, W. (2023). Seed dispersal distance classes and dispersal modes for the European flora. Global Ecology and Biogeography, 32(9), 1485-1494. https://doi.org/10.1111/geb.13712

Pistacia lentiscus

Jordano, P. (1995). Angiosperm fleshy fruits and seed dispersers: a comparative analysis of adaptation and constraints in plant-animal interactions. The American Naturalist, 145(2), 163-191. https://doi.org/10.1086/285735

Lososová, Z., Axmanová, I., Chytrý, M., Midolo, G., Abdulhak, S., Karger, D. N., ... & Thuiller, W. (2023). Seed dispersal distance classes and dispersal modes for the European flora. Global Ecology and Biogeography, 32(9), 1485-1494. https://doi.org/10.1111/geb.13712

Aronne, G., & Wilcock, C. C. (1994). First evidence of myrmecochory in fleshy-fruited shrubs of the Mediterranean region. New Phytologist, 127(4), 781-788. https://doi.org/10.1111/j.1469-8137.1994.tb02982.x

Platycladus orientalis

Liao, T., Liu, G., Guo, L., Wang, Y., Yao, Y., & Cao, J. (2021). Bud Initiation, microsporogenesis, megasporogenesis, and cone development in Platycladus orientalis. HortScience, 56(1), 85-93. https://doi.org/10.21273/HORTSCI15479-20

Our Data<br><br>Lososová, Z., Axmanová, I., Chytrý, M., Midolo, G., Abdulhak, S., Karger, D. N., ... & Thuiller, W. (2023). Seed dispersal distance classes and dispersal modes for the European flora. Global Ecology and Biogeography, 32(9), 1485-1494. https://doi.org/10.1111/geb.13712
Prunus dulcis
Colic, S., Rakonjac, V., Zec, G., Nikolic, D., & Aksic, M. F. (2012). Morphological and biochemical evaluation of selected almond [Prunus dulcis (Mill.) DA Webb] genotypes in northern Serbia. Turkish Journal of Agriculture and Forestry, 36(4), 429-438. https://doi.org/10.3906/tar-1103-50<br><br>Lososová, Z., Axmanová, I., Chytrý, M., Midolo, G., Abdulhak, S., Karger, D. N., ... & Thuiller, W. (2023). Seed dispersal distance classes and dispersal modes for the European flora. Global Ecology and Biogeography, 32(9), 1485-1494. https://doi.org/10.1111/geb.13712<br><br>Balaguer-Romano, R., Barea-Marquez, A., Ocaña-Calahorro, F. J., Gomez, J. M., Schupp, E. W., Zhang, J., & de Casas, R. R. (2021). The potential role of synzoochory in the naturalization of almond tree. Basic and Applied Ecology, 50, 97-106. https://doi.org/10.1016/j.baae.2020.11.004
Punica granatum
Khadivi, A., & Arab, M. (2021). Identification of the superior genotypes of pomegranate (Punica granatum L.) using morphological and fruit characters. Food Science & Nutrition, 9(8), 4578-4588. https://doi.org/10.1002/fsn3.2450<br><br>Lososová, Z., Axmanová, I., Chytrý, M., Midolo, G., Abdulhak, S., Karger, D. N., ... & Thuiller, W. (2023). Seed dispersal distance classes and dispersal modes for the European flora. Global Ecology and Biogeography, 32(9), 1485-1494. https://doi.org/10.1111/geb.13712
Pyrus bourgaeana

Jordano, P. (1995). Angiosperm fleshy fruits and seed dispersers: a comparative analysis of adaptation and constraints in plant-animal interactions. The American Naturalist, 145(2), 163-191. https://doi.org/10.1086/285735

Fedriani, J. M., & Delibes, M. (2009). Functional diversity in fruit-frugivore interactions: a field experiment with Mediterranean mammals. Ecography, 32(6), 983-992. https://doi.org/10.1111/j.1600-0587.2009.05925.x

Lososová, Z., Axmanová, I., Chytrý, M., Midolo, G., Abdulhak, S., Karger, D. N., ... & Thuiller, W. (2023). Seed dispersal distance classes and dispersal modes for the European flora. Global Ecology and Biogeography, 32(9), 1485-1494. https://doi.org/10.1111/geb.13712

Quercus ilex

Amimi, N., Ghouil, H., Zitouna-Chebbi, R., Joët, T., & Ammari, Y. (2023). Intraspecific variation of Quercus ilex L. seed morphophysiological traits in Tunisia reveals a trade-off between seed germination and shoot emergence rates along a thermal gradient. Annals of Forest Science, 80(1), 12. https://doi.org/10.1186/s13595-023-01179-7

Lososová, Z., Axmanová, I., Chytrý, M., Midolo, G., Abdulhak, S., Karger, D. N., ... & Thuiller, W. (2023). Seed dispersal distance classes and dispersal modes for the European flora. Global Ecology and Biogeography, 32(9), 1485-1494. https://doi.org/10.1111/geb.13712

Quercus suber

Ramírez-Valiente, J. A., Valladares, F., Gil, L., & Aranda, I. (2009). Population differences in juvenile survival under increasing drought are mediated by seed size in cork oak (Quercus suber L.). Forest Ecology and Management, 257(8), 1676-1683. https://doi.org/10.1016/j.foreco.2009.01.024

Lososová, Z., Axmanová, I., Chytrý, M., Midolo, G., Abdulhak, S., Karger, D. N., ... & Thuiller, W. (2023). Seed dispersal distance classes and dispersal modes for the European flora. Global Ecology and Biogeography, 32(9), 1485-1494. https://doi.org/10.1111/geb.13712

Rubus ulmifolius
Jordano, P. (1995). Angiosperm fleshy fruits and seed dispersers: a comparative analysis of adaptation and constraints in plant-animal interactions. The American Naturalist, 145(2), 163-191. https://doi.org/10.1086/285735<br><br>Fedriani, J. M., & Delibes, M. (2009). Functional diversity in fruit-frugivore interactions: a field experiment with Mediterranean mammals. Ecography, 32(6), 983-992. https://doi.org/10.1111/j.1600-0587.2009.05925.x<br><br>Lososová, Z., Axmanová, I., Chytrý, M., Midolo, G., Abdulhak, S., Karger, D. N., ... & Thuiller, W. (2023). Seed dispersal distance classes and dispersal modes for the European flora. Global Ecology and Biogeography, 32(9), 1485-1494. https://doi.org/10.1111/geb.13712
Silybum marianum
El-haak et al. 2015: El-haak, M. A., Atta, B. M., & Abd Rabo, F. F. (2015). Seed yield and important seed constituents for naturally and cultivated milk thistle (Silybum marianum) plants†. The Egyptian Journal of Experimental Biology (Botany), 11(2), 141Ц146. https://www.egyseb.net/index.php?mno=202092<br><br>Lososová, Z., Axmanová, I., Chytrý, M., Midolo, G., Abdulhak, S., Karger, D. N., ... & Thuiller, W. (2023). Seed dispersal distance classes and dispersal modes for the European flora. Global Ecology and Biogeography, 32(9), 1485-1494. https://doi.org/10.1111/geb.13712
Styphnolobium japonicum
Lim, T. K. (2013). Styphnolobium japonicum. In Edible Medicinal And Non-Medicinal Plants: Volume 7, Flowers (pp. 906-924). Dordrecht: Springer Netherlands. https://doi.org/10.1007/978-94-007-7395-0_74<br><br>Lososová, Z., Axmanová, I., Chytrý, M., Midolo, G., Abdulhak, S., Karger, D. N., ... & Thuiller, W. (2023). Seed dispersal distance classes and dispersal modes for the European flora. Global Ecology and Biogeography, 32(9), 1485-1494. https://doi.org/10.1111/geb.13712

Tipuana tipu
Pece, M. G., de Benítez, C. G., Acosta, M., Bruno, C., Saavedra, S., & Buvenas, O. (2010). Germinación de Tipuana tipu (Benth.) O. Kuntze (tipa blanca) en condiciones de laboratorio. Quebracho-Revista de Ciencias Forestales, 18(1-2), 5-15. http://www.redalyc.org/articulo.oa?id=48118695001<br><br>Barbosa, K. C., & Pizo, M. A. (2006). Seed rain and seed limitation in a planted gallery forest in Brazil. Restoration Ecology, 14(4), 504-515. https://doi.org/10.1111/j.1526-100X.2006.00162.x
Ulmus minor
Yücesan, Z., & Ak, N. (2021). Effects of seed morphology and growing media on germination percentage and growth of field elm (Ulmus minor Miller subsp Ulmus minor Miller subsp Ulmus minorminor). Austrian Journal of Forest Science, 138(1). https://www.cabidigitallibrary.org/doi/full/10.5555/20210376170<br><br>Lososová, Z., Axmanová, I., Chytrý, M., Midolo, G., Abdulhak, S., Karger, D. N., ... & Thuiller, W. (2023). Seed dispersal distance classes and dispersal modes for the European flora. Global Ecology and Biogeography, 32(9), 1485-1494. https://doi.org/10.1111/geb.13712
Washingtonia robusta
Coşkuner, Y., & Gökbudak, A. (2016). Dimensional specific physical properties of fan palm fruits, seeds and seed coats (Washingtonia robusta). http://dx.doi.org/10.1515/intag-2016-0004<br><br>Spennemann, D. H. (2020). Palms fanning out: a review of the ecological provisioning services provided by Washingtonia filifera and W. robusta in their native and exotic settings. Plant Ecology & Diversity, 13(3-4), 289-324. https://doi.org/10.1080/17550874.2020.1819465

Table S4. Models obtained to explain the likelihood of a fruit having its seeds predated or not (Seed predation), dispersed or not (Seed dispersal) or its pulp consumed or not (Fruit defleshing) by birds. Bayesian models were fitted using the *brms* package[8], incorporating species as random terms and accounting for the phylogenetic relationships of both bird and plant species. The strength of evidence for each fixed effect was assessed based on the 95% credible interval (95% CI) of the posterior distribution: effects were considered to have no support when the CI strongly overlapped zero, weak support when the CI barely overlapped zero or strong support (in bold) then the CI did not overlap zero. All model parameters showed good convergence, with Rhat ≤ 1.01 and effective sample sizes (ESS_bulk and ESS_tail) ≥ 500. Mean refers to the mean of the posterior distribution. Phylogenetic signals for plants and birds (λ_{plants} and λ_{birds}, respectively) and their 95% CI were estimated from the posterior distribution of the (co)variance matrices of the phylogenies included in the models. The proportion of variance explained by all model components (random and fixed effects; conditional R^2) and by fixed effects (marginal R^2) is reported. Random variance decomposition (expressed as % of variance explained) was performed using the *varde* package[9]. FB: fruit-to-bird size ratio.

	Seed predation			Fruit defleshing			Seed dispersal		
**Fixed effects**	**Mean**	**95% CI**		**Mean**	**95% CI**		**Mean**	**95% CI**	
Intercept	-2.51	-7.10	1.79	-2.80	-7.43	1.98	**-4.74**	**-8.12**	**-1.37**
FB ratio	**-2.48**	**-3.43**	**-1.57**	**3.39**	**1.83**	**4.95**	**-1.21**	**-2.02**	**-0.53**
FB ratio^2	**3.17**	**2.37**	**4.03**	**-1.13**	**-2.57**	**-0.25**			
Fruit type:									
Drupaceous	-2.62	-5.39	0.31	**4.40**	**0.75**	**7.79**	0.30	-1.43	2.07
Berry-like	-2.00	-5.09	1.33	-0.64	-4.25	2.91	**3.79**	**1.40**	**5.92**
Unripe	0.40	-0.27	1.02	-0.66	-2.22	0.95	0.12	-0.44	0.69

Swallower	**-2.99**	**-5.77**	**-0.22**	**-3.86**	**-5.43**	**-2.02**	**3.23**	**0.73**	**5.45**
FB ratio * Fruit type:									
FB ratio * Drupaceous	**-5.42**	**-7.75**	**-3.31**	**7.79**	**5.83**	**9.80**	**1.26**	**0.36**	**2.23**
FB ratio * Berry-like	**-2.88**	**-3.71**	**-2.14**	-1.27	-4.35	0.99	**1.88**	**0.93**	**2.92**
FB ratio * Unripe	**-4.46**	**-6.14**	**-2.85**	**-5.68**	**-9.13**	**-2.30**	0.14	-0.77	1.06
FB ratio * Swallower							1.31	-0.61	3.12
Fruit type * Ripeness:									
Drupaceous * Unripe	**6.14**	**5.41**	**6.90**	**-3.48**	**-5.10**	**-1.89**	**-1.10**	**-1.76**	**-0.45**
Berry-like * Unripe	**5.56**	**4.61**	**6.58**	-0.09	-3.85	3.63	**-7.08**	**-8.03**	**-6.18**
Fruit type * Feeding strategy:									
Drupaceous * Swallower							0.75	-0.13	1.65
Berry-like * Swallower							0.87	-0.33	2.07
Unripe * Swallower							**-2.89**	**-4.92**	**-1.03**
FB ratio * Fruit type * Ripeness:									
FB ratio * Drupaceous * Unripe	**-20.65**	**-23.70**	**-17.61**	**-5.62**	**-8.95**	**-2.28**	**-1.27**	**-2.48**	**-0.06**
FB ratio * Berry-like * Unripe	**9.75**	**7.48**	**12.15**	-0.03	-3.96	3.89	-1.76	-4.02	0.19
FB ratio * Fruit type * Feeding strategy:									
FB ratio * Drupaceous * Swallower							**-9.13**	**-11.06**	**-7.17**
FB ratio * Berry-like * Swallower							**8.86**	**6.23**	**11.52**
FB ratio * Unripe * Swallower							**-4.60**	**-7.63**	**-1.60**

Fruit type * Ripeness * Feeding strategy:									
Drupaceous * Unripe * Swallower							-0.19	-3.99	3.47
Berry-like * Unripe * Swallower							-1.71	-4.75	1.08
FB ratio * Fruit type * Ripeness * Feeding strategy:									
FB ratio * Drupaceous * Unripe * Swallower							0.05	-3.98	4.03
FB ratio * Berry-like * Unripe * Swallower							**-5.92**	**-9.27**	**-2.64**
**Phylogenetic signal**									
λ_{plants}	0.04	<0.01	0.15	0.15	0.05	0.31	0.01	<0.01	0.04
λ_{birds}	0.04	0.01	0.10	<0.01	<0.01	0.02	0.02	<0.01	0.05
**Variance explained**									
marginal R^2	0.28	0.07	0.54	0.41	0.06	0.53	0.50	0.20	0.67
conditional R^2	0.83	0.83	0.83	0.67	0.66	0.68	0.75	0.75	0.75
**Random variance decomposition**									
Bird species	<0.01	<0.01	4.11	<0.01	<0.01	2.64	<0.01	<0.01	2.51
Plant species	16.40	3.06	34.74	<0.01	<0.01	10.61	<0.01	<0.01	7.01
Bird phylogeny	0.10	0.03	0.39	0.01	<0.01	0.08	0.05	0.01	0.17
Plant phylogeny	<0.01	<0.01	0.60	0.41	0.18	1.50	<0.01	<0.01	0.13

[8]Bürkner, P. (2017). brms: An R package for Bayesian multilevel models. Journal of Statistical Software, 80(1). https://doi.org/10.18637/jss.v080.i01.

[9]Girard J, Simmons A (2025). varde: Variance Decomposition Functions. R package version 0.0.1, https://github.com/affcomlab/varde

Table S5. Total number of fruits in which birds predated seeds (Total), along with the number of those fruits in which at least some seeds were ultimately dispersed (Disp. fruits) and the corresponding estimated number of seeds dispersed (Disp. seeds). The latter was calculated by multiplying the mean number of seeds per fruit for each plant species (Table S2) by the proportion of each fruit that was consumed or transported in the bird's beak without destroying the seed. The associated dispersal mechanism (i.e., endozoochory, epizoochory or stomatochory) is also indicated (Dispersal mechanism).

Plant species	Fruit type	Total	Disp. fruits	Disp. seeds	Dispersal mechanism
Arbutus unedo	Berry-like	10	6	61.92	endozoochory
Campsis radicans	Dry	154	48	20044.8	stomatochory
Catalpa bignonioides	Dry	78	2	150.56	stomatochory
Ceratonia siliqua	Dry	92	10	76	stomatochory
Cercis siliquastrum	Dry	150	9	46.2	stomatochory
Cupressus sempervirens	Dry	28	10	1186.22	stomatochory
Ficus microcarpa	Berry-like	99	18	3124.8	endozoochory, stomatochory
Magnolia grandiflora	Dry	125	4	205.38	stomatochory, epizoochory
Melia azedarach	Drupaceous	326	7	20.22	stomatochory
Morus alba	Berry-like	530	100	606.84	endozoochory, stomatochory
Morus nigra	Berry-like	236	30	432	endozoochory
Myrtus communis	Berry-like	161	78	262.08	endozoochory, stomatochory, epizoochory
Platycladus orientalis	Dry	158	3	603.6	stomatochory, epizoochory
Punica granatum	Berry-like	16	1	2	epizoochory

Plant species	Fruit type	Total	Disp. fruits	Disp. seeds	Dispersal mechanism
Pyrus bourgaeana	Drupaceous	36	6	30.02	stomatochory
Silybum marianum	Dry	359	61	7320	stomatochory
Styphnolobium japonicum	Drupaceous	792	32	100.8	stomatochory

Table S6. Models obtained to explain the likelihood of a fruit having its seeds dispersed or not by endozoochory, stomatochory or epizoochory. Bayesian models were fitted using *brms* package[8], incorporating species as random terms and accounting for the phylogenetic relationships of both bird and plant species. The strength of evidence for each fixed effect was assess based on the 95% credible interval (95% CI) of the posterior distribution: effects were considered to have no support when the CI strongly overlapped zero, weak support when the CI barely overlapped zero or strong support (in bold) when the CI did not overlap zero. All model parameters showed good convergence, with Rhat ≤ 1.01 and effective sample sizes (ESS_bulk and ESS_tail) ≥ 660. Mean refers to the mean of the posterior distribution. Phylogenetic signals for plants and birds (λ_{plants} and λ_{birds}, respectively) and their 95% CI were estimated from the posterior distribution of the (co)variance matrices of the phylogenies included in the models. The proportion of variance explained by all model components (random and fixed effects; conditional R^2) and by fixed effects (marginal R^2) is reported. Random variance decomposition (expressed as % of variance explained) was performed using the *varde* package[9]. FB: fruit-to-bird size ratio.

	**Endozoochory**			**Stomatochory**			**Epizoochory**		
**Fixed effects**	**Mean**	**95% CI**		**Mean**	**95% CI**		**Mean**	**95% CI**	
Intercept	**-6.71**	**-11.19**	**-2.31**	1.23	-3.20	5.36	-4.00	-8.23	0.83
FB ratio	-0.51	-1.97	1.02	0.29	-1.13	1.68	0.09	-2.25	1.90
FB ratio^2	**-1.95**	**-4.22**	**-0.07**	**2.09**	**0.31**	**4.24**	**-1.26**	**-3.30**	**-0.04**
Fruit type:									
Drupaceous	1.68	-0.87	4.18	-1.04	-3.71	1.48	-1.94	-4.98	1.08
Berry-Like	**4.02**	**0.76**	**6.91**	**-4.10**	**-6.98**	**-0.72**	**2.91**	**0.12**	**5.49**
Swallower	0.75	-2.21	3.55	-0.68	-3.51	2.18			

FB ratio^2 * Fruit type:									
FB ratio^2 * Drupaceous	-2.65	-5.32	0.08	**3.20**	**0.54**	**5.96**			
FB ratio^2 * Berry-Like	**2.57**	**0.68**	**4.83**	**-2.46**	**-4.69**	**-0.60**			
FB ratio^2 * Swallower	1.45	-0.98	3.86	-1.65	-4.05	0.75			
Fruit type * Feeding strategy:									
Drupaceous * Swallower	**3.54**	**1.29**	**5.80**	**-3.95**	**-6.24**	**-1.67**			
Berry-Like * Swallower	-0.67	-2.91	1.54	1.22	-1.02	3.55			
FB ratio^2 * Fruit type * Feeding strategy:									
FB ratio^2 * Drupaceous * Swallower	**2.99**	**0.25**	**5.83**	**-3.37**	**-6.21**	**-0.51**			
FB ratio^2 * Berry-Like * Swallower	-1.29	-3.77	1.20	1.37	-1.05	3.83			
**Phylogenetic signal**									
λ_{plants}	0.03	<0.01	0.07	0.03	<0.01	0.09	0.04	0.02	0.08
λ_{birds}	0.02	<0.01	0.09	0.02	<0.01	0.08	0.06	0.05	0.14
**Variance explained**									
marginal R^2	0.24	0.01	0.48	0.38	0.06	0.56	0.27	<0.01	0.50
conditional R^2	0.59	0.59	0.60	0.66	0.65	0.66	0.14	0.10	0.18
**Random variance decomposition**									
Bird species	1.30	0.17	7.97	2.05	0.59	8.60	<0.01	<0.01	11.99
Plant species	<0.01	<0.01	6.39	<0.01	<0.01	9.79	<0.01	<0.01	4.80
Bird phylogeny	<0.01	<0.01	0.34	<0.01	<0.01	0.28	0.01	0.04	4.14

Plant phylogeny	0.05	<0.01	0.27	<0.01	<0.01	0.33	<0.01	<0.01	0.44

[8]Bürkner, P. (2017). brms: An R package for Bayesian multilevel models. Journal of Statistical Software, 80(1). https://doi.org/10.18637/jss.v080.i01

[9]Girard J, Simmons A (2025). varde: Variance Decomposition Functions. R package version 0.0.1, https://github.com/affcomlab/varde

Table S7. Number of individuals of each bird species performing one, two or all interaction types (predation, defleshing and/or dispersal) and dispersal mechanisms (endozoochory, stomatochory and/or epizoochory) during focal observations (of up to 5 minutes).

Bird species	Interaction types			Dispersal mechanisms		
	One	Two	All	One	Two	All
Carduelis carduelis	95	0	0	2	0	0
Chloris chloris	336	24	0	46	0	0
Coloeus monedula	31	6	0	36	0	0
Columba livia	60	0	0	21	0	0
Columba palumbus	74	0	0	30	0	0
Curruca communis	8	0	0	6	0	0
Curruca melanocephala	69	1	0	44	1	0
Cyanopica cyanus	317	72	0	251	55	0
Fringilla coelebs	46	0	0	1	0	0
Linaria cannabina	21	0	0	3	0	0
Myiopsitta monachus	615	49	1	132	51	1
Oriolus oriolus	10	0	0	10	0	0
Passer domesticus	272	18	0	119	2	0
Passer hispaniolensis	70	0	0	12	0	0
Passer montanus	7	0	0	2	0	0

Pica pica	83	4	0	73	2	0
Psittacula krameri	1536	311	4	620	105	5
Serinus serinus	34	0	0	2	0	0
Streptopelia decaocto	78	1	0	6	0	0
Sturnus unicolor	417	6	0	337	61	0
Sylvia atricapilla	436	22	0	295	6	0
Sylvia borin	181	0	0	169	0	0
Thectocercus acuticaudatus	15	0	0	0	0	0
Turdus merula	610	21	0	564	38	0
Turdus philomelos	56	0	0	54	0	0

Table S8. Models obtained to explain the likelihood of an individual bird displaying or not continuum or combinations of different interaction types with plants (seed predation, seed dispersal and/or pulp defleshing) and polychory (seed dispersal by endozoochory, stomatochory and/or epizoochory) during focal observations (up to five minutes). Bayesian models were fitted using *brms* package[8] incorporating species as random terms and accounting for the phylogenetic relationships of both bird and plant species. The strength of evidence for each fixed effect was assess based on the 95% credible interval (95% CI) of the posterior distribution: effects were considered to have no support when the CI strongly overlapped zero, weak support when the CI barely overlapped zero or strong support (in bold) when the CI did not overlap zero. All model parameters showed good convergence, with Rhat ≤ 1.01 and effective sample sizes (ESS_bulk and ESS_tail) ≥ 500. Mean refers to the mean of the posterior distribution. Phylogenetic signals for plants and birds (λ_{plants} and λ_{birds}, respectively) and their 95% CI were estimated from the posterior distribution of the (co)variance matrices of the phylogenies included in the models. The proportion of variance explained by all model components (random and fixed effects; conditional R^2) and by fixed effects (marginal R^2) is reported. Random variance decomposition (expressed as % of variance explained) was performed using the *varde* package[9]. FB: fruit-to-bird size ratio.

	**Continuum**			**Polychory**		
**Fixed effects**	**Mean**	**95% CI**		**Mean**	**95% CI**	
Intercept	**-4.25**	**-6.91**	**-1.42**	**-5.71**	**-9.07**	**-2.19**
FB ratio	**-2.41**	**-3.93**	**-1.09**	**-1.25**	**-2.18**	**-0.39**
Foraging time	**0.21**	**0.10**	**0.33**	**0.50**	**0.34**	**0.66**
Unripe	**1.21**	**0.17**	**2.29**			
Fruit type:						
Drupaceous	1.18	-0.29	2.69	0.96	-1.34	3.36

Berry-Like	-0.76	-2.66	1.06	2.66	-0.01	5.06
FB ratio * Fruit type:						
FB ratio * Drupaceous	0.98	-0.73	2.69			
FB ratio * Berry-like	-0.52	-2.98	1.90			
FB ratio * Unripe	**2.48**	**0.52**	**4.42**			
Fruit type * Ripeness:						
Drupaceous * Unripe	0.00	-1.26	1.22			
Berry-like * Unripe	**3.02**	**1.27**	**4.85**			
FB ratio * Fruit type * Ripeness:						
FB ratio * Drupaceous * Unripe	1.69	-0.91	4.24			
FB ratio * Berry-like * Unripe	**-3.91**	**-6.77**	**-1,07**			
**Phylogenetic signal**						
λ_{plants}	<0.01	<0.01	0.02	0.01	<0.01	0.04
λ_{birds}	<0.01	<0.01	0.02	0.02	0.01	0.07
**Variance explained**						
marginal R^2	0.09	<0.01	0.33	0. 09	<0.01	0.40
conditional R^2	0.17	0.14	0.33	0.24	0.21	0.28
**Random variance decomposition**						
Bird species	<0.01	<0.01	4.12	<0.01	<0.01	6.78
Plant species	<0.01	<0.01	3.73	<0.01	<0.01	5.41

Bird phylogeny	<0.01	<0.01	0.08	<0.01	<0.01	0.25
Plant phylogeny	<0.01	<0.01	0.06	<0.01	<0.01	0.13

[8]Bürkner, P. (2017). brms: An R package for Bayesian multilevel models. Journal of Statistical Software, 80(1). https://doi.org/10.18637/jss.v080.i01.

[9]Girard J, Simmons A (2025). varde: Variance Decomposition Functions. R package version 0.0.1, https://github.com/affcomlab/varde

Table S9. Number of individuals of each plant species on which birds were observed performing one, two or all interaction types (predation, defleshing and/or dispersal) and dispersal mechanism (i.e., endozoochory, stomatochory and/or epizoochory) during focal observations (30 minutes).

Plant species	Interaction types			Dispersal mechanisms		
	One	Two	All	One	Two	All
Arbutus unedo	5	2	0	6	1	0
Campsis radicans	0	6	0	6	0	0
Catalpa bignonioides	7	2	0	2	0	0
Celtis australis	17	17	0	14	7	0
Ceratonia siliqua	3	3	0	4	0	0
Cercis siliquastrum	2	7	0	7	0	0
Citrus ^{x} aurantium	9	4	0	4	0	0
Cupressus sempervirens	3	4	0	4	0	0
Eriobotrya japonica	7	4	0	4	0	0
Feijoa sellowiana	2	0	0	0	2	0
Ficus carica	19	0	0	5	9	5
Ficus microcarpa	8	3	0	1	6	4
Fraxinus angustifolia	11	1	0	1	0	0
Fraxinus excelsior	4	2	0	2	0	0

Helianthus annuus	13	0	0	0	0	0
Lantana ˣ *strigocamara*	4	1	5	6	4	0
Ligustrum japonicum	4	9	0	8	1	0
Livistona chinensis	0	15	0	4	11	0
Magnolia grandiflora	1	3	0	2	1	0
Melia azedarach	9	11	4	10	8	0
Morus alba	36	12	0	12	24	9
Morus nigra	17	8	0	3	14	3
Myrtus communis	2	6	0	6	1	1
Olea europaea	3	12	0	9	3	0
Olea europaea europaea	2	20	2	11	10	0
Phoenix canariensis	12	26	4	19	15	0
Phoenix dactylifera	15	12	3	14	0	0
Pistacia lentiscus	10	5	0	15	0	0
Platycladus orientalis	3	1	0	0	1	0
Prunus dulcis	1	7	0	7	0	0
Punica granatum	5	2	0	3	0	0
Pyrus bourgaeana	6	8	2	9	0	0
Quercus ilex	7	6	7	17	0	0
Quercus suber	1	3	0	4	0	0

Rubus ulmifolius	2	0	0	2	0	0
Silybum marianum	13	4	0	4	0	0
Styphnolobium japonicum	9	11	2	12	2	0
Tipuana tipu	3	1	0	1	0	0
Ulmus minor	13	1	0	1	0	0
Washingtonia robusta	6	16	0	8	12	0

Table S10. Models obtained to explain the likelihood of an individual plant experiencing continuum or combinations of different interaction types (seed predation, seed dispersal and/or pulp defleshing) with birds and polychory (seed dispersed by endozoochory, stomatochory and/or epizoochory) during focal observations (30 minutes). Bayesian models were fitted using *brms* package[8] incorporating species as random terms and accounting for their phylogenetic relationships. The strength of evidence for each fixed effect was assess based on the 95% credible interval (95% CI) of the posterior distribution: effects were considered to have no support when the CI strongly overlapped zero, weak support when the CI barely overlapped zero or strong support (in bold) when CI did not overlap zero. All model parameters showed good convergence, with Rhat ≤ 1.01 and effective sample sizes (ESS_bulk and ESS_tail) ≥ 500. Mean refers to the mean of the posterior distribution. The phylogenetic signal (λ_{plants}) and its 95% CI was estimated from the posterior distribution of the (co)variance matrice of the phylogeny included in the models. The proportion of variance explained by all model components (random and fixed effects; conditional R^2) and by fixed effects (marginal R^2) is reported. Random variance decomposition (expressed as % of variance explained) was performed using the *varde* package[9]. FB: fruit-to-bird size ratio.

	**Continuum**			**Polychory**		
**Fixed effects**	**Mean**	**95% CI**		**Mean**	**95% CI**	
Intercept	-0.32	-3.28	2.11	**-3.31**	**-5.64**	**-1.15**
Mean FB ratio	0.56	-0.34	1.46	**-1.06**	**-1.51**	**-0.65**
Mean FB ratio^2	**-1.01**	**-1.80**	**-0.28**			
Cumulative foraging time	**0.43**	**0.04**	**0.83**	-0.08	-0.33	0.17
Number of consumers	**0.47**	**0.04**	**0.92**	**0.48**	**0.22**	**0.76**
Diversity of consumers	**0.64**	**0.32**	**0.98**	-0.06	-0.31	0.19

Ripeness:						
Both stages	**2.66**	**1.08**	**4.55**			
Unripe	0.17	-0.47	0.80			
Fruit type:						
Drupaceous	0.63	-0.85	2.05	0.80	-0.93	2.65
Berry-Like	-1.36	-3.21	0.55	**1.95**	**0.01**	**3.77**
**Phylogenetic signal**						
λ_{plants}	0.01	<0.01	0.02	<0.01	<0.01	0.01
**Variance explained**						
marginal R^2	0.22	0.06	0.32	0.12	<0.01	0.27
conditional R^2	0.39	0.34	0.42	0.19	0.14	0.23
**Random variance decomposition**						
Plant species	<0.01	<0.01	5.12	<0.01	<0.01	3.39
Plant phylogeny	<0.01	<0.01	0.07	<0.01	<0.01	0.05

[8]Bürkner, P. (2017). brms: An R package for Bayesian multilevel models. Journal of Statistical Software, 80(1). https://doi.org/10.18637/jss.v080.i01

[9]Girard J, Simmons A (2025). varde: Variance Decomposition Functions. R package version 0.0.1, https://github.com/affcomlab/varde

Table S11. Percentage of early unripe fruits (i.e., they had not formed seeds that could ripen after partial consumption; Barnett et al. 2012; Blanco et al. 2016) that were predated by birds during focal observations (30 minutes). In all cases, their immature seeds were predated exclusively by parrot species (i.e., by *P. krameri*, *M. monachus* and *T. acuticaudatus*). Percentages are calculated relative to the total number of consumed fruits. **N** refers to the total number of early unripe fruits handled by parrots on each plant species. References are shown in the main text.

**Plant species**	**Fruit type**	**% early unripe fruits**	**N**
Celtis australis	Drupaceous	6.64	108
Morus alba	Berry-like	6.16	123
Phoenix canariensis	Drupaceous	6.9	41
Phoenix dactylifera	Drupaceous	2.61	24
Tipuana tipu	Dry	19.27	16

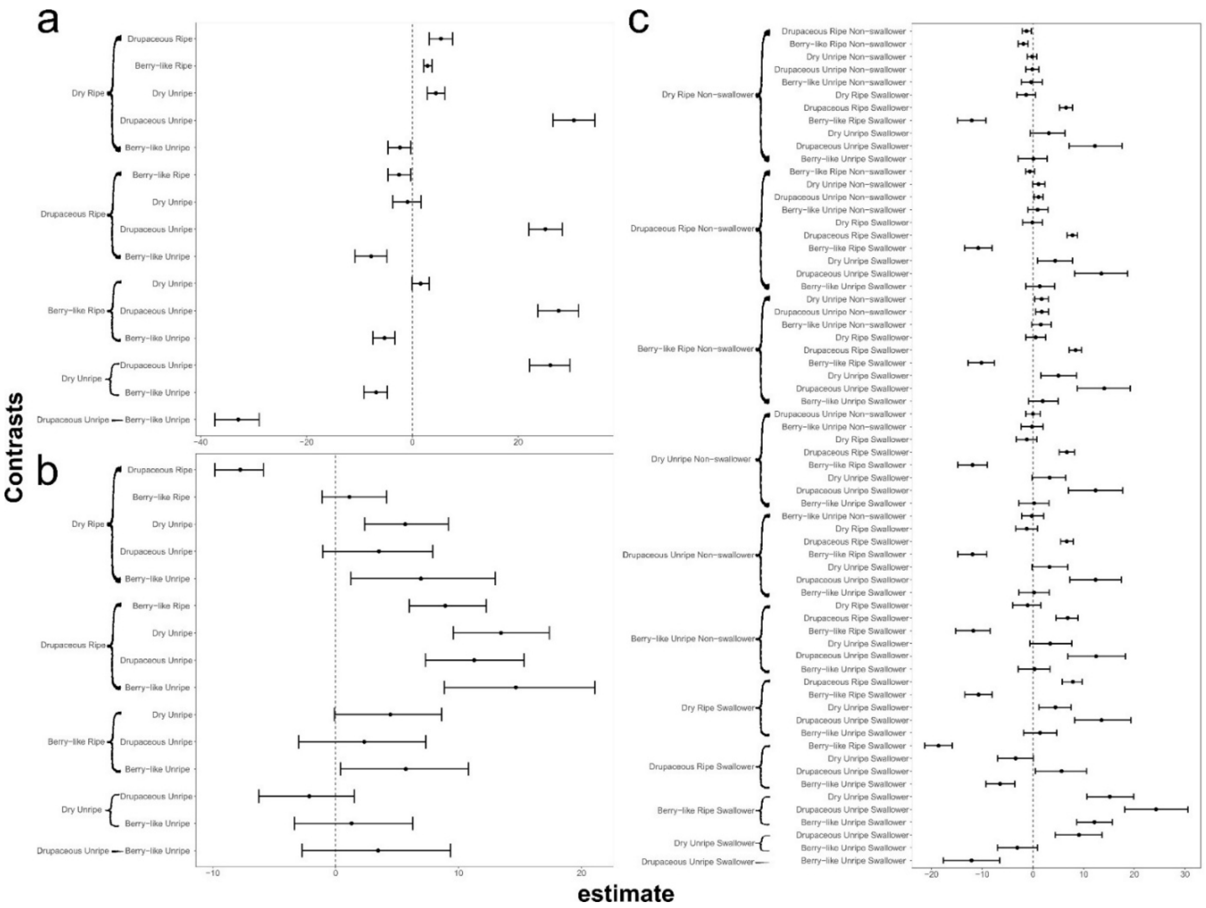

Figure S1. Interactive effects of fruit type, ripeness and bird feeding strategy on the probability of seed predation (a), fruit defleshing (b), and seed dispersal (c). Marginal means and associated 95% credible intervals were estimated from the models presented in Tables S6 and S7, using the *emtrends* function from the *emmeans* package (Lenth, 2019)[10].

[10]Lenth R (2025). emmeans: Estimated Marginal Means, aka Least-Squares Means. R package version 1.11.1-00001, https://rvlenth.github.io/emmeans/

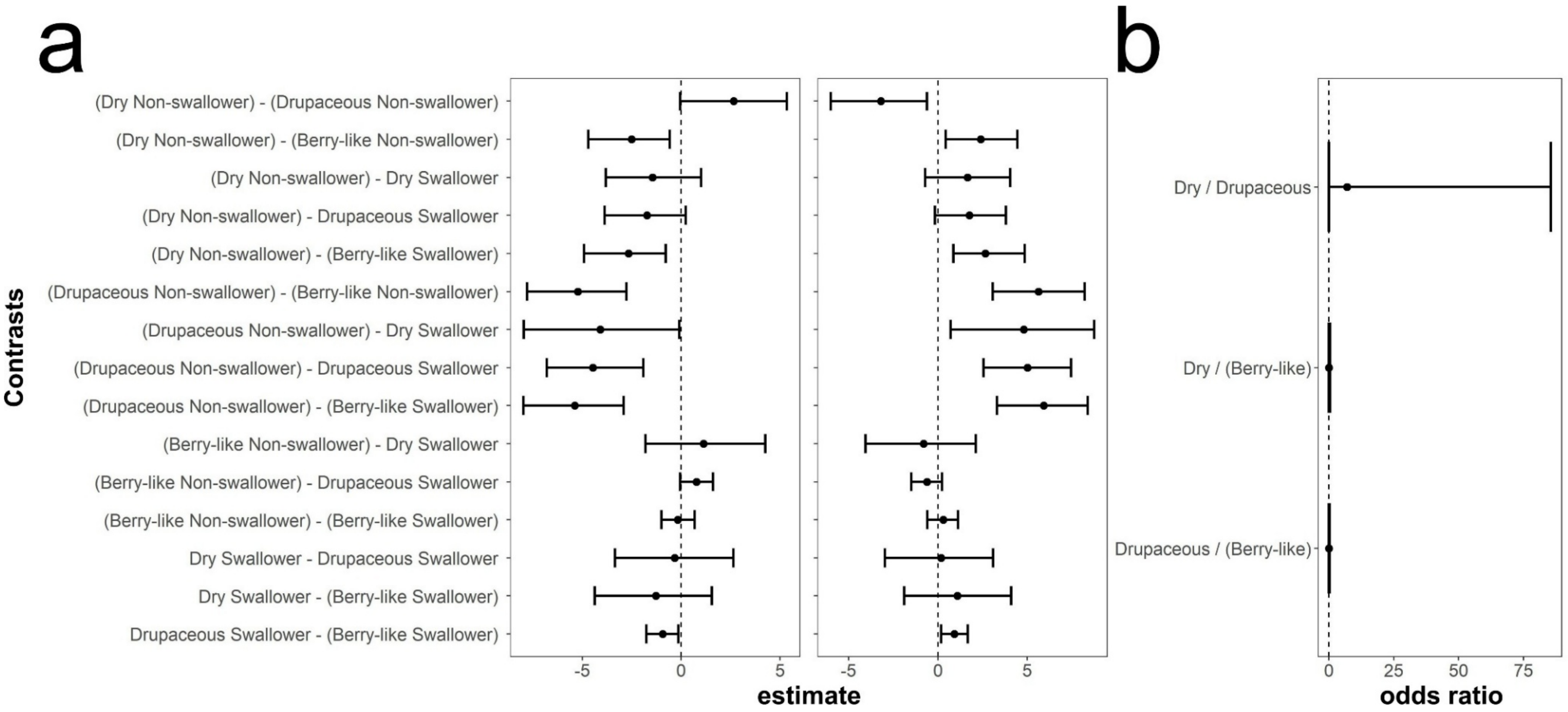

Figure S2. a) Interactive effects of fruit type and bird feeding strategy on the probability of seeds dispersal by endozoochory (left plot) and stomatochory (right plot). Marginal means and associated 95% credible intervals were estimated from the models presented in Tables S6 and S7, using the *emtrends* and *emmeans* functions from the *emmeans* package (Lenth, 2019)[10]. b) Relative likelihood (odds ratio) of dispersal by epizoochory of dry, drupaceous and berry-like fruits. Values above 1 indicate a higher probability of epizoochory compared to the reference fruit type (second term), while values below 1 indicate a lower probability compared to the reference fruit type.

[10]Lenth R (2025). emmeans: Estimated Marginal Means, aka Least-Squares Means. R package version 1.11.1-00001, https://rvlenth.github.io/emmeans/

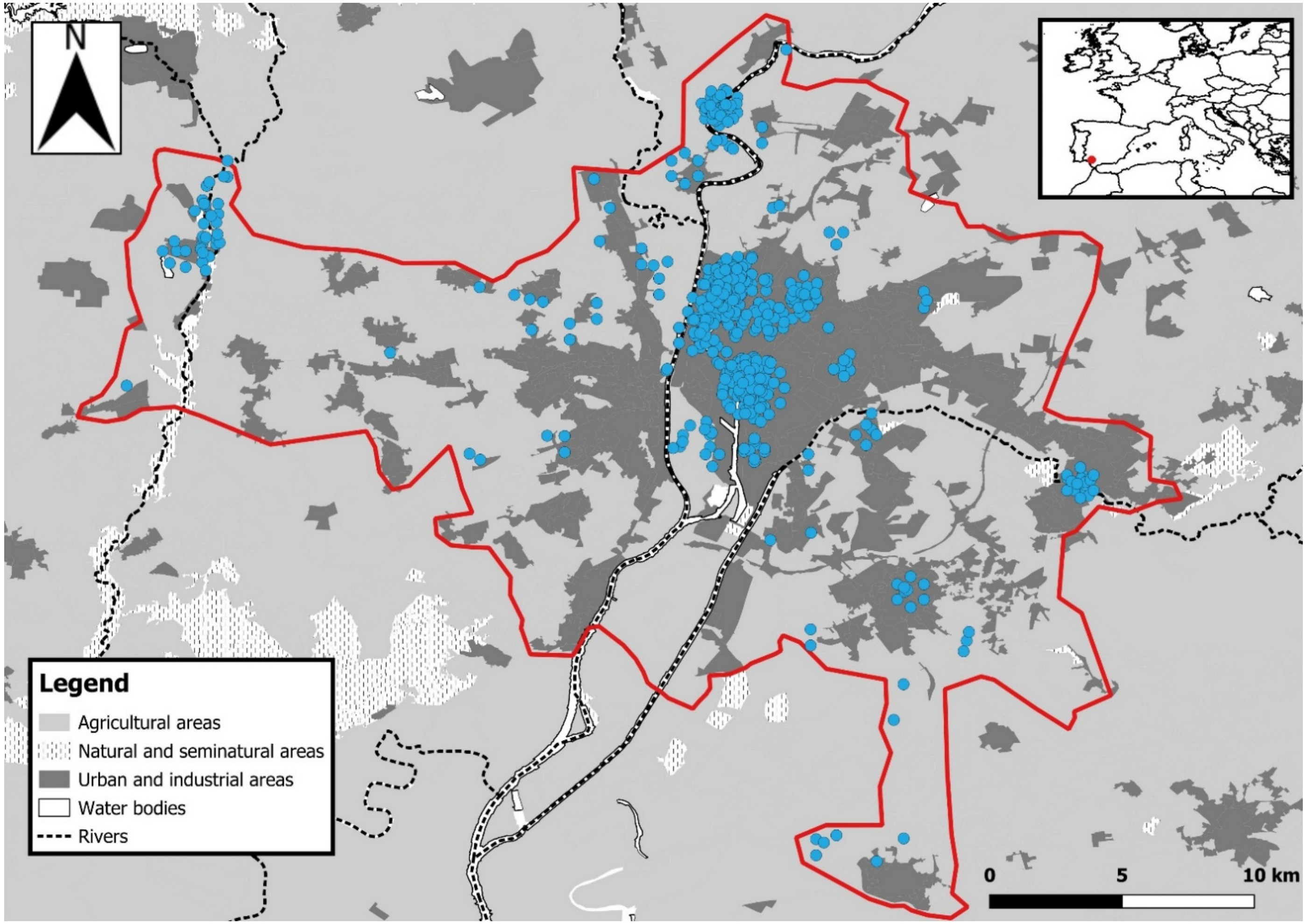

Figure S3. Map of the study area (outlined in red), encompassing the metropolitan area of Seville (southern Spain) and its surroundings (indicated by a red dot in the inset map). Blue dots represent the locations of monitored plants where plant-bird interactions and dispersal mechanisms were recorded (Izquierdo-Palma et al., 2025).
