# Supplementary material for: Context-dependent plant–bird interactions shape polychory across the antagonism–mutualism continuum
Source: Commun Biol. 2026 Apr 28;9:739. doi: 10.1038/s42003-026-10142-x (PMC13226708; doi:10.1038/s42003-026-10142-x)
Supplement: Supplementary file 2 — Description of Additional Supplementary Files [file 42003_2026_10142_MOESM2_ESM.pdf]

Description of Additional Supplementary File

File name: Supplementary Data 1
Description: Global dataset. For each plant species, the proportion of fruits associated with different plant-bid interaction types and seed dispersal mechanisms for each bird species.

File name: Supplementary Data 2
Description: Percentage of intact seeds in faecal samples and regurgitated pellets from different bird species recorded in the study area between 2015 and 2023.

File name: Supplementary Data 3
Description: Percentage of intact seeds dispersed by different zoochorous mechanisms and recorded in perches, nesting and roosting areas of study birds.
