# Supplementary material for: Context-dependent plant–bird interactions shape polychory across the antagonism–mutualism continuum
Source: Commun Biol. 2026 Apr 28;9:739. doi: 10.1038/s42003-026-10142-x (PMC13226708; doi:10.1038/s42003-026-10142-x)
Supplement: Supplementary file 6 — Reporting Summary [file 42003_2026_10142_MOESM6_ESM.pdf]

nature portfolio

Corresponding author(s): Dailos Hernández-Brito

Last updated by author(s): 2026/04/02

Reporting Summary

Nature Portfolio wishes to improve the reproducibility of the work that we publish. This form provides structure for consistency and transparency in reporting. For further information on Nature Portfolio policies, see our Editorial Policies and the Editorial Policy Checklist.

Statistics

For all statistical analyses, confirm that the following items are present in the figure legend, table legend, main text, or Methods section.

n/a	Confirmed	
☐	☒	The exact sample size (*n*) for each experimental group/condition, given as a discrete number and unit of measurement
☐	☒	A statement on whether measurements were taken from distinct samples or whether the same sample was measured repeatedly
☐	☒	The statistical test(s) used AND whether they are one- or two-sided<br>*Only common tests should be described solely by name; describe more complex techniques in the Methods section.*
☐	☒	A description of all covariates tested
☐	☒	A description of any assumptions or corrections, such as tests of normality and adjustment for multiple comparisons
☐	☒	A full description of the statistical parameters including central tendency (e.g. means) or other basic estimates (e.g. regression coefficient) AND variation (e.g. standard deviation) or associated estimates of uncertainty (e.g. confidence intervals)
☐	☒	For null hypothesis testing, the test statistic (e.g. F, t, r) with confidence intervals, effect sizes, degrees of freedom and P value noted<br>*Give P values as exact values whenever suitable.*
☐	☒	For Bayesian analysis, information on the choice of priors and Markov chain Monte Carlo settings
☒	☐	For hierarchical and complex designs, identification of the appropriate level for tests and full reporting of outcomes
☐	☒	Estimates of effect sizes (e.g. Cohen's *d*, Pearson's *r*), indicating how they were calculated

Our web collection on statistics for biologists contains articles on many of the points above.

Software and code

Policy information about availability of computer code

Data collection	No software was used.
Data analysis	R Core Team. R: A language and environment for statistical computing. R Foundation for Statistical Computing, Vienna (2021).

For manuscripts utilizing custom algorithms or software that are central to the research but not yet described in published literature, software must be made available to editors and reviewers. We strongly encourage code deposition in a community repository (e.g. GitHub). See the Nature Portfolio guidelines for submitting code & software for further information.

Data

Policy information about availability of data

All manuscripts must include a data availability statement. This statement should provide the following information, where applicable:

- Accession codes, unique identifiers, or web links for publicly available datasets
- A description of any restrictions on data availability
- For clinical datasets or third party data, please ensure that the statement adheres to our policy

All data are included in the paper and the Supplementary Material.

Research involving human participants, their data, or biological material

Policy information about studies with human participants or human data. See also policy information about sex, gender (identity/presentation), and sexual orientation and race, ethnicity and racism.

Reporting on sex and gender	This information has not been collected.
Reporting on race, ethnicity, or other socially relevant groupings	This information has not been collected.
Population characteristics	This information has not been collected.
Recruitment	This information has not been collected.
Ethics oversight	This information has not been collected.

Note that full information on the approval of the study protocol must also be provided in the manuscript.

Field-specific reporting

Please select the one below that is the best fit for your research. If you are not sure, read the appropriate sections before making your selection.

☐ Life sciences ☐ Behavioural & social sciences ☒ Ecological, evolutionary & environmental sciences

For a reference copy of the document with all sections, see nature.com/documents/nr-reporting-summary-flat.pdf

Ecological, evolutionary & environmental sciences study design

All studies must disclose on these points even when the disclosure is negative.

Study description	We assessed plant–bird interactions along the continuum from antagonism to mutualism, as well as across different zoochorous mechanisms, using Bayesian generalised linear mixed models. To achieve this, we quantified these interactions by monitoring fruiting tree and shrub species throughout an entire annual cycle.
Research sample	lant diversity in the study area was too vast to monitor comprehensively. We therefore conducted preliminary work to identify the main plant species utilized by birds for feeding, thus preselecting 40 species from 18 families. These selected species formed the basis for constructing the ecological networks and included native Mediterranean and exotic ornamental species with arboreal and shrub-like growth habits.<br>A total of 25 bird species were identified while they were foraging in selected plant species (Order Columbiformes: Columba livia, C. palumbus, Streptopelia decaocto; Order Passeriformes: Coloeus monedula, Cyanopica cooki, Pica pica, Oriolus oriolus, Sturnus unicolor, Sylvia atricapilla, S. borin, Curruca communis, C. melanocephala, Turdus merula, T. philomelos, Passer domesticus, P. hispaniolensis, P. montanus, Linaria cannabina, Carduelis carduelis, Chloris chloris, Fringilla coelebs, and Serinus serinus; and Order Psittaciformes: Myiopsitta monachus, Psittacula krameri, Thectocercus acuticaudatus).
Sampling strategy	Over the course of a full annual cycle, we recorded bird-fruit interactions weekly. To maximise sampling while minimising pseudoreplication, each plant was GPS-recorded and observed for 30 minutes at each stage of its fruit phenology (unripe and ripe), beginning when foraging activity by birds was detected. During these sessions, foraging individuals were observed for 5 minutes, and all interactions with fruits were recorded. Monitoring occurred during the first three hours after sunrise and the last three hours before sunset, coinciding with the peak of bird foraging activity, while avoiding suboptimal weather conditions such as strong wind or rain. To maximize detection of interactions, we monitored as many individuals of each plant species as possible, covering the entire study area.
Data collection	We systematically documented avian foraging behavior over a full annual cycle, recording interactions between birds and fruiting plants, along with the associated seed dispersal mechanisms. Each plant was monitored for 30 minutes by a team of 3–4 observers, who closely followed individual foraging birds for up to five minutes. Observations were conducted from distances of 10–50 meters using optical equipment (10×42 binoculars and 20×60 spotting scopes) to minimize disturbance. Observers included the authors: D.H.-B., F.H., J.I.-P., J.L.T., and M.C.
Timing and spatial scale	Over the course of a full annual cycle (April 2022 and May 2023), we recorded plant-bird interactions weekly.
Data exclusions	Interactions in which birds engaged exclusively with early-unripe fruits, primarily involving seed predation, were excluded from the model analyses.
Reproducibility	All attempts to repeat the experiment were successfull.
Randomization	To maximise sampling while minimising pseudoreplication, each plant was GPS-recorded and observed for 30 minutes at each stage of its fruit phenology (unripe and ripe), beginning when foraging activity by birds was detected. To maximize detection of interactions, we monitored as many individuals of each plant species as possible, covering the entire study area.

Blinding | Blinding was not relevant for our study.

Did the study involve field work? ☒ Yes ☐ No

Field work, collection and transport

Field conditions	Monitoring occurred during the first three hours after sunrise and the last three hours before sunset, coinciding with the peak of bird foraging activity, while avoiding suboptimal weather conditions such as strong wind or rain.
Location	Seville (Spain).
Access & import/export	Not needed.
Disturbance	Not disturbance.

Reporting for specific materials, systems and methods

We require information from authors about some types of materials, experimental systems and methods used in many studies. Here, indicate whether each material, system or method listed is relevant to your study. If you are not sure if a list item applies to your research, read the appropriate section before selecting a response.

Materials & experimental systems

n/a	Involved in the study	
☒	☐	Antibodies
☒	☐	Eukaryotic cell lines
☒	☐	Palaeontology and archaeology
☐	☒	Animals and other organisms
☒	☐	Clinical data
☒	☐	Dual use research of concern
☐	☒	Plants

Methods

n/a	Involved in the study	
☒	☐	ChIP-seq
☒	☐	Flow cytometry
☒	☐	MRI-based neuroimaging

Animals and other research organisms

Policy information about studies involving animals; ARRIVE guidelines recommended for reporting animal research, and Sex and Gender in Research

Laboratory animals	The study did not involve laboratory animals.
Wild animals	Each plant was GPS-recorded and observed for 30 minutes at each stage of its fruit phenology (unripe and ripe), beginning when foraging activity by birds was detected. During these sessions, foraging individuals were observed for 5 minutes, and all interactions with fruits and seeds were recorded.
Reporting on sex	Birds were not sexed visually.
Field-collected samples	We collected fecal samples and regurgitated pellets from various bird species observed within the study area. Since our primary objective was to quantify the percentage of intact seeds present in these samples, we did not establish parameters related to sample preservation or apply any post-identification experimental protocols.
Ethics oversight	No ethical approval was needed as the study was observational.

Note that full information on the approval of the study protocol must also be provided in the manuscript.

Dual use research of concern

Policy information about dual use research of concern

Hazards

Could the accidental, deliberate or reckless misuse of agents or technologies generated in the work, or the application of information presented in the manuscript, pose a threat to:

No	Yes	
☒	☐	Public health
☒	☐	National security
☒	☐	Crops and/or livestock
☒	☐	Ecosystems
☒	☐	Any other significant area

Experiments of concern

Does the work involve any of these experiments of concern:

No	Yes	
☒	☐	Demonstrate how to render a vaccine ineffective
☒	☐	Confer resistance to therapeutically useful antibiotics or antiviral agents
☒	☐	Enhance the virulence of a pathogen or render a nonpathogen virulent
☒	☐	Increase transmissibility of a pathogen
☒	☐	Alter the host range of a pathogen
☒	☐	Enable evasion of diagnostic/detection modalities
☒	☐	Enable the weaponization of a biological agent or toxin
☒	☐	Any other potentially harmful combination of experiments and agents

Plants

Seed stocks

Between 2015 and 2023, we collected seeds resulting from two distinct foraging processes exhibited by different bird species within the study area: (1) seeds found in fecal samples and regurgitated pellets (endozoochory), and (2) seeds dropped beneath perches after being transported away from the parent plant (mainly stomatochory). As our primary goal was to quantify the percentage of intact seeds within these samples, we did not record data on sample preservation or implement any experimental procedures following seed identification.

Novel plant genotypes

Not applicable.

Authentication

Seeds were identified to the finest possible taxonomic level (species or genus) using a dissecting microscope and taxonomic keys from the Flora Ibérica guide (Castroviejo et al., 1986).
